# Spectral dynamics of guided edge removals and identifying transient amplifiers for death–Birth updating

**DOI:** 10.1007/s00285-023-01937-1

**Published:** 2023-06-07

**Authors:** Hendrik Richter

**Affiliations:** Faculty of Engineering, HTWK Leipzig University of Applied Sciences, Leipzig, Germany

**Keywords:** Evolutionary dynamics, Transient amplifiers, Regular graphs, Laplacian spectra, Edge manipulation, 92D15, 05C90, 05C50, 05C75, 94C15

## Abstract

The paper deals with two interrelated topics: (1) identifying transient amplifiers in an iterative process, and (2) analyzing the process by its spectral dynamics, which is the change in the graph spectra by edge manipulation. Transient amplifiers are networks representing population structures which shift the balance between natural selection and random drift. Thus, amplifiers are highly relevant for understanding the relationships between spatial structures and evolutionary dynamics. We study an iterative procedure to identify transient amplifiers for death–Birth updating. The algorithm starts with a regular input graph and iteratively removes edges until desired structures are achieved. Thus, a sequence of candidate graphs is obtained. The edge removals are guided by quantities derived from the sequence of candidate graphs. Moreover, we are interested in the Laplacian spectra of the candidate graphs and analyze the iterative process by its spectral dynamics. The results show that although transient amplifiers for death–Birth updating are generally rare, a substantial number of them can be obtained by the proposed procedure. The graphs identified share structural properties and have some similarity to dumbbell and barbell graphs. We analyze amplification properties of these graphs and also two more families of bell-like graphs and show that further transient amplifiers for death–Birth updating can be found. Finally, it is demonstrated that the spectral dynamics possesses characteristic features useful for deducing links between structural and spectral properties. These feature can also be taken for distinguishing transient amplifiers among evolutionary graphs in general.

## Introduction

Evolution occurs by natural selection and genetic drift. Thus, if a mutant arises in a population of residents, its evolutionary dynamics is affected by the mutant’s fitness (as this influences the mutant’s chances in natural selection) and drift (which basically is understood as a random process). The balance between natural selection and random drift may vary over different spatial population structures. There are some spatial structures which amplify natural selection, thus shifting the balance towards the influence of fitness. Some other spatial structures suppress natural selection, thus reversing the effect. A mathematical understanding of the relationships between spatial structures and evolutionary dynamics is highly relevant for real biological processes, as for instance shown for cancer initiation and progression (Hindersin et al. 2016; Komarova et al. 2003; Komarova 2006; Nowak et al. 2003; Vermeulen et al. 2013), ageing of tissues (Cannataro et al. 2016, 2017), spread of infections (Ottino-Löffler et al. 2017a, b) and microbial evolution of antibiotic resistance (Krieger et al. 2020).

Spatial structures can be interpreted as a network and modelled mathematically as a graph. Networks amplifying or suppressing selection have been intensively studied in the past decades (Adlam et al. 2015; Alcalde Cuesta et al. 2017; Allen et al. 2020; Hindersin and Traulsen 2015; Hindersin et al. 2019; Jamieson-Lane and Hauert 2015; McAvoy and Allen 2021; Monk 2018; Pavlogiannis et al. 2017, 2018; Tkadlec et al. 2020, 2021). The ability of a network to amplify (or suppress) selection not only depends on the spatial structure, but also on other factors. One factor is where in the network and under what circumstances mutation occurs in the first place. Most previous works (Hindersin and Traulsen 2015; Jamieson-Lane and Hauert 2015; Lieberman et al. 2005; Monk et al. 2014; Möller et al. 2019; Pavlogiannis et al. 2017) have assumed that heritable mutations mainly occur in adult individuals with the same probability over time. For the network this means mutations equally likely appear at all vertices, which is called uniform initialization. As an alternative, we may assume mutations to occur mainly in new offspring, which means in the network mutations appear more likely at vertices more frequently replaced, which is called temperature initialization (Adlam et al. 2015; Allen et al. 2021; Pavlogiannis et al. 2018; Tkadlec et al. 2019). Recently, it has been shown that at least for Birth–death updating amplification properties vary over initialization (Allen et al. 2021).

Another factor is the updating mechanism by which mutants and/or residents propagate on the graph. Two mechanism frequently studied are Birth–death (Bd) and death–Birth (dB) updating. For Bd updating many graphs represent population structures with amplification properties. For instance, by analyzing a large number of random graphs with $N\le 14$ vertices an extensive numerical study identified a multitude of amplifiers of selection for Bd (Hindersin and Traulsen 2015). More recently, a systematic study checking all graphs up to $N\le 10$ vertices found that almost all graphs have amplification properties (Allen et al. 2021).

For dB updating we find the opposite. The mentioned study (Hindersin and Traulsen 2015) analyzing a larger number of random graphs found no amplifiers for dB updating. This prompted the assumption that either amplifiers for dB are very rare, at least for graphs with small orders, or even there are none. Meanwhile, theoretical and numerical works have modified and partly corrected this view. On the one hand, it has been shown that for dB updating universal amplification is not possible (Tkadlec et al. 2020). At most, an evolutionary graph can be a transient amplifier (meaning the amplification only takes place for a certain range of fitness). In addition, a method has been devised which allows computing with polynomial time complexity if for weak selection a graph is a (tangential) amplifier (Allen et al. 2020). The method is based on calculating the coalescence times of random walks (Allen et al. 2017) on the graph and finding the vertex with the largest remeeting time. If a single edge from this vertex is removed, with some likelihood the resulting graph is a transient amplifier. The method also implies identifying transient amplifiers for dB updating by edge removals from regular graphs taken as an input to the method. Recent results have shown that for a small but non-negligible fraction of all pairwise non-isomorphic regular graphs with certain order and degree transient amplifiers are obtained by such a single edge removal (Richter 2021).

However, the resulting graphs are close to the regularity of the input graphs and typically only for cubic and quartic regular graphs transient amplifiers have been identified. In this paper we study how transient amplifiers for dB updating can be obtained by multiple edge removals embedded in an iterative algorithmic process. As this gives a larger variety of transient amplifiers, particularly with a stronger perturbation to the regularity of the input graphs, a more profound analysis of structural and spectral properties of amplification can be done. A main tool in this analysis is spectral dynamics, which is concerned with changes in the graph spectra over graph manipulations (Chen and Zhang 2017; Zhang et al. 2009). We here study the spectral dynamics of the normalized (and standard) Laplacian spectra over edge removals. Our main result is that although transient amplifiers for dB updating are relatively rare, a significant number of them can be identified by the iterative method. For instance, for graphs on $N=11$ and $N=12$ vertices, for all existing degrees there are regular input graphs which can be disturbed into amplifiers by guided edge removals. This also applies for graphs on $N=\{14,20,26\}$ vertices with degree $k=N-3$. The results also show that transient amplifiers for dB updating identified by the iterative process share certain structural properties. They mainly consist of two cliques of highly (frequently completely) connected vertices which are joined by bridges of one or two edges. Thus, these graphs have some similarity to barbell and dumbbell graphs (Ghosh et al. 2008; Wang et al. 2009). The analysis of the spectral dynamics also reveals shared spectral characteristics. Interlacing results state that the Laplacian spectra generally shrink by edge removals (Atay and Tuncel 2014; Chen et al. 2004; van den Heuvel 1995). By analyzing the algebraic connectivity as well as the smoothed spectral density of the whole spectrum, it can be shown that edge removal processes leading to transient amplifiers can be distinguished from processes not leading to amplifiers. This opens up the possibility to link structural and spectral properties of transient amplifiers. The results also add to answering a fundamental mathematical question in graph theory which is the relationships between graph spectra and graph structure.

## Methods

### Identifying transient amplifiers

We study an evolving population of *N* individuals whose spatial structure is described by an undirected (and unweighted) graph $\mathcal {G}=(V,E)$. Each individual is associated with a vertex $v_i \in V$ and an edge $e_{ij}\in E$ indicates that the individuals placed on $v_i$ and $v_j$ are neighbors (Allen et al. 2017; Lieberman et al. 2005; Ohtsuki et al. 2007; Pattni et al. 2015; Richter 2017). The graph $\mathcal {G}$ is simple and connected, and each vertex $v_i$ has degree $k_i$. Thus, there is no self-replacement and an individual on $v_i$ has $k_i$ neighbors.

With residents and mutants there are two types of individuals. Residents have a constant fitness specified to unity, while mutants’ fitness is $r>0$. An individual’s type can go from resident to mutant (and back) by a fitness-dependent selection process. We study a death–birth (dB) process, e.g. (Allen et al. 2017, 2020; Pattni et al. 2015). An individual is chosen uniformly at random and dies, thus vacating the vertex it occupied. One of the neighbors is selected to give birth with a probability depending on its fitness. The neighbor selected transfers its type and thus replaces the dead individual. To indicate that birth in such an updating process is fitness-dependent, but death is not, we write dB updating, as suggested by (Hindersin and Traulsen 2015).

We consider uniform initialization and define the fixation probability $\varrho _{\mathcal {G}}$ as the expected probability that for a single mutant appearing at a vertex uniformly at random finally all vertices of the graph $\mathcal {G}$ become the mutant type. We are particularly interested in how the fixation probability $\varrho _{\mathcal {G}}(r)$ is related to the fixation probability $\varrho _{\mathcal {N}}(r)$ of the complete graph with *N* vertices for varying fitness *r*. We categorize the graphs as follows (Adlam et al. 2015; Allen et al. 2020; Hindersin and Traulsen 2015; Pavlogiannis et al. 2017). A graph $\mathcal {G}$ is called an amplifier of selection if $\varrho _{\mathcal {G}}(r)<\varrho _{\mathcal {N}}(r)$ for $0<r<1$ and $\varrho _{\mathcal {G}}(r)>\varrho _{\mathcal {N}}(r)$ for $r>1$. A suppressor of selection is characterized by $\varrho _{\mathcal {G}}(r)>\varrho _{\mathcal {N}}(r)$ for $0<r<1$ and $\varrho _{\mathcal {G}}(r)<\varrho _{\mathcal {N}}(r)$ for $r>1$. Finally, we have a transient amplifier if $\varrho _{\mathcal {G}}(r)<\varrho _{\mathcal {N}}(r)$ for $r_{\min }<r<1$ and $r>r_{\max }$, and also there is $\varrho _{\mathcal {G}}(r)>\varrho _{\mathcal {N}}(r)$ for $1<r<r_{\max }$ and some $0<r_{\min }<1<r_{\max }<\infty$.

The structural and spectral analysis as well as the iterative procedure is based on three recent results on amplifiers for dB. First, it has been shown that universal amplification is not achievable for dB updating and only transient amplification is possible (Tkadlec et al. 2020). Second, a numerical test has been proposed for weak selection (where $r=1+\delta$ and $\delta \rightarrow 0$). The test can be executed with polynomial time complexity and allows to detect if a graph $\mathcal {G}$ is a (tangential) amplifier (Allen et al. 2020). Third, the test has been applied to checking all regular graphs up to a certain order and degree. It was shown that a single edge removal produces transient amplifiers for a small but non-negligible number of cubic and quartic regular graphs. Moreover, a spectral analysis has demonstrated that there is a close relationship between the Laplacian spectra and amplification (Richter 2021).

The numerical test identifying transient amplifiers considers coalescing random walks (Allen et al. 2017) and requires computing the effective population size $N_\textrm{eff}$ from the relative degree $\pi _i=k_i/\sum _{j \in \mathcal {G}} k_j$ and the remeeting time $\tau _i$ of vertex $v_i$ (Allen et al. 2020). The coalescence times $\tau _{ij}$ are obtained from solving a system of $$\left( {\begin{matrix}N\\ 2\end{matrix}}\right) $$ linear equations

1 $$\begin{aligned} \tau _{ij}= \left\{ \begin{array}{ll} 0 &{} \quad i=j\\ 1+ \frac{1}{2} \sum _{k \in \mathcal {G}} (p_{ik}\tau _{jk}+p_{jk}\tau _{ik}) &{} \quad i\ne j \end{array} \right. ,\end{aligned}$$

where $p_{ij}=e_{ij}/k_i$ are the step probabilities $p_{ij}=1/k_i$, if $e_{ij}=1$ and $p_{ij}=0$, otherwise. From these coalescence times $\tau _{ij}$ the remeeting time $\tau _i$ is calculated by

2 $$\begin{aligned} \tau _i= 1+ \sum _{j \in \mathcal {G}} p_{ij}\tau _{ij}. \end{aligned}$$

Remeeting times obey the identity condition

3 $$\begin{aligned} \sum _{i \in \mathcal {G}} \pi _{i} ^2\tau _{i}^{}=1. \end{aligned}$$

A graph $\mathcal {G}$ is an amplifier of weak selection if (Allen et al. 2020)

4 $$\begin{aligned} N_\textrm{eff}= \sum _{i \in \mathcal {G}} \pi _i \tau _i >N. \end{aligned}$$

An amplifier of weak selection can be identified by the following perturbation method. For a *k*-regular graph $\mathcal {G}$, we have $k_i=k$ and $\pi _i=1/N$ for all $i=1,2,\ldots ,N$. Thus, with the identity condition (3), we obtain from Eq. (4):

5 $$\begin{aligned} N_\textrm{eff}= \sum _{i \in \mathcal {G}} \tau _i/N=N \sum _{i \in \mathcal {G}} \pi _{i} ^2\tau _{i}^{}=N. \end{aligned}$$

The equality $N_\textrm{eff}=N$ in Eq. (5) indicates that *k*-regular graphs cannot be amplifiers of weak selection, which is also a consequence of the isothermal theorem (Lieberman et al. 2005). But disturbing the regularity may possibly change the equality to $N_\textrm{eff}>N$. Moreover, such a perturbation is most promising if we remove an edge from the vertex $v_i$. This is the vertex of the *k*-regular graph to be tested with the largest remeeting time $\tau _i$, that is $\max (\tau _i)={\max }_{i \in \mathcal {G}} \, \tau _i$. The argument is as follows. If we take a regular graph and cause a small perturbation by removing an edge, the relative degree $\pi _i$ and the remeeting time $\tau _i$ undergo small deviations $\varDelta \pi _i$ and $\varDelta \tau _i$. The perturbation for the effective population size, Eq. (4), is

6 $$\begin{aligned} \varDelta N_\textrm{eff}= \sum _{i \in \mathcal {G}} \varDelta (\pi _i \tau _i) \approx \sum _{i \in \mathcal {G}} (\varDelta \pi _i \tau _i+\pi _i \varDelta \tau _i), \end{aligned}$$

while from the identity condition (3) we obtain

7 $$\begin{aligned} \sum _{i \in \mathcal {G}} \varDelta \left( \pi _{i} ^2\tau _{i}^{}\right) \approx \sum _{i \in \mathcal {G}} \left( 2 \pi _i \varDelta \pi _{i} \tau _{i}^{}+ \pi _{i} ^2 \varDelta \tau _{i}^{}\right) \approx 0. \end{aligned}$$

Since for the unperturbed regular graph $\pi _i=1/N$ holds, we get from Eq. (7)

8 $$\begin{aligned} 1/N \sum _{i \in \mathcal {G}} \varDelta \tau _{i}^{} \approx -2 \sum _{i \in \mathcal {G}} \varDelta \pi _{i} \tau _{i}^{}. \end{aligned}$$

Inserting Eq. (8) into Eq. (6) and still observing $\pi _i=1/N$ for the unperturbed regular graph yields

9 $$\begin{aligned} \varDelta N_\textrm{eff} \approx -\sum _{i \in \mathcal {G}} \varDelta \pi _i \tau _i. \end{aligned}$$

The relationship (9) suggests that a positive perturbation of the effective population size (and thus the possibility to get $N_\textrm{eff}>N$) can be achieved if for a large $\tau _i$ the perturbation entails a decrease of the relative degree $\pi _i$, which means a negative $\varDelta \pi _i$. Thus, Eq. (9) can be interpreted as a procedure to identify transient amplifiers. We need to find the vertex $v_{n_1}$, $n_1= \arg {\max }_{i \in \mathcal {G}} \, \tau _i$, with the largest remeeting time and proceed by removing each of the *k* edges adjacent to the vertex, thus obtaining *k* candidate graphs. Possibly there is a transient amplifier among these candidate graphs, which can be tested by condition (4).

We may repeat this perturbation by removing another edge and thus introduce second (subsequent) deviations $\varDelta ^2 \pi _i$ and $\varDelta ^2 \tau _i$ to the relative degree $\pi _i$ and the remeeting time $\tau _i$. Analogously to Eqs. (6) and (7) we obtain for the identify condition (3) and the effective population size (4) the following second perturbations:

10 $$\begin{aligned} \sum _{i \in \mathcal {G}} \varDelta ^2 \left( \pi _{i} ^2\tau _{i}^{}\right) \approx \sum _{i \in \mathcal {G}}\left( 2 \pi _i \varDelta ^2 \pi _{i} \tau _{i}^{}+ 2\left( \varDelta \pi _i \right) ^2 \tau _i+4\pi _i \varDelta \pi _i \varDelta \tau _i+\pi _{i} ^2 \varDelta ^2 \tau _{i}^{}\right) \approx 0. \end{aligned}$$

and

11 $$\begin{aligned} \varDelta ^2 N_\textrm{eff}= \sum _{i \in \mathcal {G}} \varDelta ^2\left( \pi _i \tau _i\right) \approx \sum _{i \in \mathcal {G}} \left( \varDelta ^2 \pi _i \tau _i+2\varDelta \pi _i \varDelta \tau _i +\pi _i \varDelta ^2 \tau _i \right) . \end{aligned}$$

Combining these equations and using $\pi _i=1/N$ yields

12 $$\begin{aligned} \varDelta ^2 N_\textrm{eff}\approx -\sum _{i \in \mathcal {G}} \left( \left[ \varDelta ^2 \pi _i +2N\left( \varDelta \pi _i \right) ^2\right] \tau _i+2\varDelta \pi _i \varDelta \tau _i\right) \end{aligned}$$

with $\varDelta \tau _i=\tau _i(1)-\tau _i(0)$. Also this equation can be interpreted as a calculating instruction to obtain a transient amplifier with a positive $\varDelta ^2 N_\textrm{eff}$, but in addition to the effect of $\tau _i$ (as in Eq. (9)), we now also have the influence of $\varDelta \tau _i$. In other words, we may remove another edge from the vertex $v_{n_1}$ with the largest $\tau _i$. But the effect of a negative $\varDelta ^2 \pi _i$ is countered by $2N\left( \varDelta \pi _i \right) ^2 >0$, which means we need $|\varDelta ^2 \pi _i| >2N\left( \varDelta \pi _i \right) ^2$ for the largest $\tau _i$ to become effective. Alternatively, or even additionally, we may remove an edge from the vertex $v_{n_2}$, $n_2= \arg {\max }_{i \in \mathcal {G}} \, \varDelta \tau _i$, with the largest $\varDelta \tau _i$.

For the perturbation procedure repeated a third time, we get

13 $$\begin{aligned} \varDelta ^3 N_\textrm{eff}&\approx -\sum _{i \in \mathcal {G}} \Big ( \left[ \varDelta ^3 \pi _i +6N\varDelta ^2 \pi _i \varDelta \pi \right] \tau _i \nonumber \\&\quad + \left[ 3 \varDelta ^2 \pi _i + 6N\left( \varDelta \pi _i \right) ^2\right] \varDelta \tau _i +3\varDelta \pi _i \varDelta ^2 \tau _i \Big ) \end{aligned}$$

with $\varDelta ^2 \tau _i=\tau _i(2)-2\tau _i(1)+\tau _i(0)$. The instruction for identifying transient amplifiers associated with the third perturbation involves either to remove another edges from $v_{n_1}$ and/or $v_{n_2}$, or to remove an edge from $v_{n_3}$, $n_3= \arg {\max }_{i \in \mathcal {G}} \, \varDelta ^2 \tau _i$, with the largest $\varDelta ^2 \tau _i$, or any combination of removals from the edge set $(v_{n_1},v_{n_2},v_{n_3})$.

We may continue to perturb the graph by further edge removals and obtain for the $(j+1)$-th perturbation of $N_\textrm{eff}$ caused by removing the $(j+1)$-th edge from a regular graph

14 $$\begin{aligned} \varDelta ^{j+1} N_\textrm{eff}\approx -\sum _{i \in \mathcal {G}} \left( \sum _{p=0}^{j-1} \left( c_p(N,\varDelta ^q \pi _i) \cdot \varDelta ^p \tau _i \right) + (j+1) \varDelta \pi _i \varDelta ^j \tau _i \right) \end{aligned}$$

with the coefficients $c_p(N,\varDelta ^q \pi _i)$ depending on *N* and $\varDelta ^q \pi _i$, $0 \le q \le j$, $\varDelta ^0 \tau _i=\tau _i$ and $$\varDelta ^j\tau _i= \sum _{p=0}^j (-1)^{j-p} \left( {\begin{matrix}j\\ p\end{matrix}}\right) \tau _i(p)$$ is the forward difference, see Eqs. (12) and (13) for $j=1$ and $j=2$. Such a general calculating instruction suggests an iterative procedure to identify transient amplifiers starting with a regular input graph. The iterative procedure is presented in Algorithm 1. Its basic form is an enumerative, brute-force search. Additional steps for an approximative, greedy search are denoted in italics and parenthesis. We next discuss features and properties of the algorithm.

The input to Algorithm 1 is a regular graph $\mathcal {G}_{in}$ with degree *k*. It can be taken from the set of all simple connected pairwise non-isomorphic *k*-regular graphs on *N* vertices with degree $k\ge 3$, whose number of known for small *N*, see e.g. (Meringer 1999; Richter 2021; WolframMathWorld 2022). As discussed in the Sect. 3, not all regular graphs can be disturbed into transient amplifiers. In Algorithm 1 a graph is denoted by $\mathcal {G}$ and may belong to a set of graphs $\mathbb {G}$ with $|\mathbb {G}|$ indicating the number of graphs in the graph set. The “remaining graphs” in the for-loops of line 6–8 and line 21–23 are the graphs created by the edge removals minus the graphs that became disconnected by the removal or are isomorphic to another graph in the set. From the regular input graph *k* edges can be removed from each vertex which gives *k* candidate graphs for the next iteration. After the first edge removal any given vertex has $\kappa \le k$ edges, which gives $\kappa$ candidate graphs.
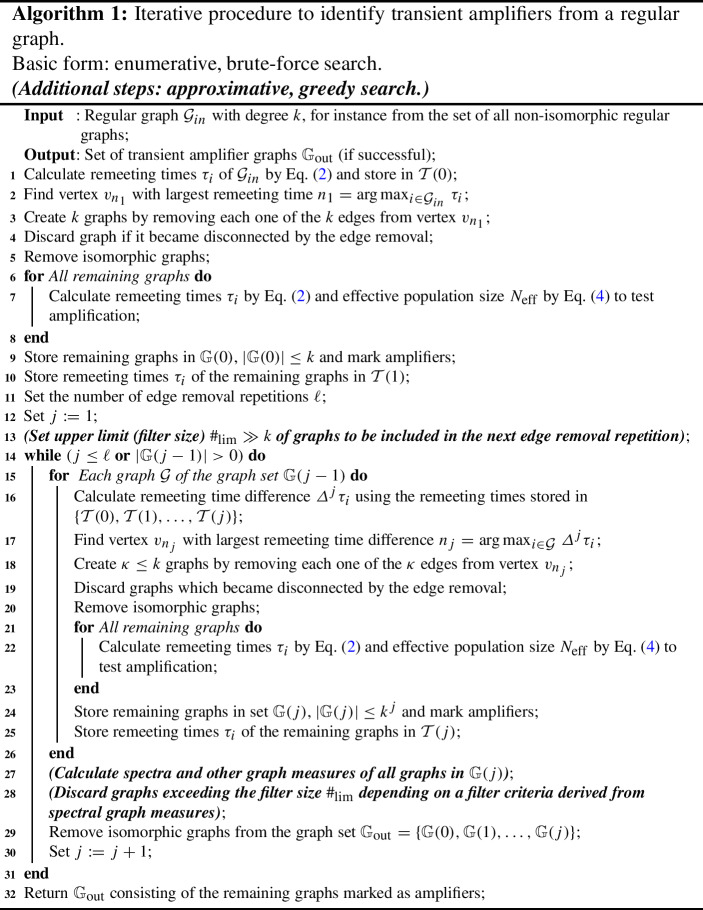


If from a given regular graph we repeat to remove edges, then sooner or later the graph will become disconnected. Thus, an important parameter of the algorithm is the number of allowed edge removal repetitions $\ell$. A regular graph has $\frac{kN}{2}$ edges and a connected graph has at least $N-1$ edges, which gives us an upper bound of edge removals: $1+\frac{(k-2)N}{2}$. Consequently, the number of edge removals $\ell$ may vary between $1 \le \ell \le 1+\frac{(k-2)N}{2}$. This bound is for the total number of edges to be removed from the graph. From a given vertex at most *k* edges can be removed before the vertex is no longer connected to the remainder of the graph. If an edge removal disconnects the graph, Algorithm 1 discards the graph. In other words, in order to keep a graph as a potential structure to be perturbed into a transient amplifier, we should not disconnect it by a needless edge removal. Although suggested by Eqs. (12)–(14) as a possibility, we thus should sparsely (if at all) remove additional edges from the vertex $v_{n_1}$, which is the vertex with the largest initial remeeting time. In the implementation of Algorithm 1, additional edges from $v_{n_1}$ are only removed if there is $v_{n_j}=v_{n_1}$ for $1<j\le \ell$. The same constraint (reducing the number of instances where edges are removed from the same vertex) also applies to the edge set $(v_{n_2},v_{n_3},\ldots ,v_{n_{j-1}})$. Thus, the vertex $v_{n_j}$ from which the *j*-th edge is removed by Algorithm 1 is solely determined by $n_{j}= \arg {\max }_{i \in \mathcal {G}} \, \varDelta ^j \tau _i$.

Algorithm 1 in its basic form is a breadth-first search with a brute-force enumeration of all possible (non-isomorphic) graphs resulting from iterative edge removals. It also relies substantially upon identifying isomorphic graphs. Roughly speaking, isomorphism means that two graphs are structurally alike and merely differ in how the vertices and edges are named. More precisely, two graphs are isomorphic if there is a bijective mapping between their vertices which preserves adjacency (Bondy and Murty 2008), pp. 12–14. Unfortunately, the computational problem of finding out whether or not two finite graphs are isomorphic is not solvable in polynomial time (Arvind and Torán 2005; Babai 2019), which is a major limitation to the applicability of Algorithm 1. Therefore, we substitute detecting isomorphic graphs by detecting cospectral graphs, which is computationally less expensive. The rationale of using cospectral as a proxy for isomorphic is that all isomorphic graphs are cospectral. On the other hand, cospectral graphs can be non-isomorphic. Thus, we might discard graphs which could possibly have been additional sources of transient amplifiers. However, numerical studies suggest that non-isomorphic pairs of graphs with the same spectrum are not very frequent and the effect of mistaking cospectral for non-isomorphic can be minimized by using the spectrum of the normalized Laplacian (Butler and Grout 2011).

Another limitation of Algorithm 1 in its basic form is the exponential growth of the number of candidate graphs produced by iterative edge removals, which restricts the applicability to small *k* and $\ell$. However, the transient amplifiers produced for small *k* and $\ell$ mostly have only a small perturbation to their input regularity and degree distribution. Thus, if we also want to study transient amplifiers with possibly stronger perturbations to their regularity and more unbalanced degree distributions, larger *k* and $\ell$ would be desirable. To counter the growth of the number of candidate graphs and achieve practical computability, we need to modify the basic form of Algorithm 1. Therefore, we set a limit $\#_{\mathcal {G}} \gg k$ to the number of graphs to be included in the next iteration. This restricts the number of graphs taken as an input to the subsequent repetition of edge removals and thus bounds the exponential growth of the number of candidate graphs. In this paper it is suggested to evaluate spectral graph measures to decide which graphs (if the limit $\#_{\mathcal {G}}$ is exceeded) are included in the next iteration. In some sense, the modifications to the basic form of the algorithm work like a filter which passes only a limited number of graphs selected by their spectral properties. Thus, we call the limit $\#_{\mathcal {G}}$ the filter size. The modified Algorithm 1 is a kind of approximative, greedy search for finding transient amplifiers of death–Birth updating. In Algorithm 1 the additional steps augmenting the basic form are given in italics and parenthesis.

### Graph spectra and edge removals

An (undirected and unweighted) graph $\mathcal {G}=(V,E)$ is specified algebraically by a symmetric adjacency matrix $A=\{a_{ij}\}$ with $a_{ij}=a_{ji}=1$ indicating that the vertices $v_i$ and $v_j$ are connected by the edge $e_{ij} \in E$. With the vertex degree $k_i=\sum _{j=1}^{N} a_{ij}$, we additionally get a degree matrix $D=\textrm{diag}$ $(k_1,k_2,\ldots ,k_N)$. For the spectral analysis of an evolutionary graph $\mathcal {G}$ we take *A* and *D*, and consider the standard Laplacian $L_{\mathcal {G}}=D-A$ and the normalized Laplacian $\varLambda _{\mathcal {G}}=I-D^{-1/2}AD^{-1/2}$. The spectrum of the standard Laplacian is denoted by $\mu (\mathcal {G})$ and consists of *N* eigenvalues $0=\mu _1\le \mu _2 \le \cdots \le \mu _N$, while for the spectrum of the normalized Laplacian we have $\lambda (\mathcal {G})$ with $0=\lambda _1\le \lambda _2 \le \cdots \lambda _N\le 2$. The second smallest eigenvalues $\mu _2$ and $\lambda _2$ are frequently called algebraic connectivity. From the Laplacian eigenvalues a spectral distance *d* can be defined which is useful for comparing two families (or classes) of graphs $(\mathcal {G})$ and $(\mathcal {G}')$. If each family only contains a single member, we can also compare two graphs $\mathcal {G}$ and $\mathcal {G}'$. Therefore, we consider a smoothed spectral density which convolves the eigenvalues $\lambda _i$ with a Gaussian kernel with standard deviation $\sigma$ (Banerjee and Jost 2009; Banerjee 2012; Gu et al. 2016)

15 $$\begin{aligned} \varphi _{\mathcal {G}}(x)= \frac{1}{N}\sum _{i=1}^{N} \frac{1}{\sqrt{2 \pi \sigma ^2}} \exp {\left( \frac{(x-\lambda _i)^2}{2 \sigma ^2} \right) }. \end{aligned}$$

We set $\sigma =1/(3N)$. From this continuous spectral density we can define a pseudometric on graphs by the distance (Gu et al. 2016)

16 $$\begin{aligned} d(\mathcal {G},\mathcal {G}^{\prime })= \int _0^2|\varphi _{\mathcal {G}}(x)-\varphi _{\mathcal {G}'}(x)|\hbox {d}x. \end{aligned}$$

Equations (15) and (16) are defined for the normalized Laplacian spectrum $\lambda (\mathcal {G})$. The spectrum is bounded in the interval [0, 2] for any graph order and degree and can thus be easily compared for variable order and degree. However, the spectral density and the spectral distance can also be defined for the standard Laplacian spectrum $\mu$ by replacing $\lambda _i$ by $\mu _i$ in Eq. (15), albeit with a variable upper integration limit in Eq. (16).

Suppose we have a graph $\mathcal {G}=(V,E)$ and remove one of its edges. Thus, the vertex set is preserved but the edge set is changed. We use $(\mathcal {G}-e_{ij})$ for denoting the graph resulting from the edge $e_{ij} \in E$ being removed from $\mathcal {G}$. As discussed in the previous section, for the greedy algorithm we need spectral measures for deciding which graphs should be included in the subsequent repetition of edge removals. We next review some results about edge removals and spectral characteristics useful for directing the greedy algorithm towards finding transient amplifiers.

Several interlacing results connect graph spectra with edge removals (Atay and Tuncel 2014; Chen et al. 2004; van den Heuvel 1995). For the spectra of the standard Laplacian there is $\mu _{i-1}(\mathcal {G}) \le \mu _i(\mathcal {G}-e_{ij}) \le \mu _{i}(\mathcal {G})$, $i=2,3,\ldots ,N$. This particularly means for the algebraic connectivity (the second smallest eigenvalue), we have always a positive spectral shift $\mu _2(\mathcal {G})-\mu _{2}(\mathcal {G}-e_{ij})=\alpha$ with $\alpha \ge 0$. For the normalized Laplacian, the eigenvalue interlacing is $\lambda _{i-1}(\mathcal {G}) \le \lambda _i(\mathcal {G}-e_{ij}) \le \lambda _{i+1}(\mathcal {G})$, $i=2,3,\ldots ,N-1$. Consequently, eigenvalue interlacing differs between the standard Laplacian and the normalized Laplacian. The eigenvalues of the standard Laplacian decrease or remain unchanged if an edge is removed, while for the normalized Laplacian the eigenvalues may in fact also increase. Decreasing or increasing of the algebraic connectivity associated with the normalized Laplacian is bounded by $0< \lambda _2(\mathcal {G}-e_{ij}) \le \lambda _3(\mathcal {G})$. The increase of $\lambda _2$ related to an edge removal is also known as Braess’s paradox (Eldan et al. 2017).

## Results

### Computational setup

In the previous section an algorithmic process has been derived and analyzed which identifies transient amplifiers of death–Birth updating by employing an iterative design procedure. We next discuss an application of the algorithm. The algorithm has two variants. It can be either an enumerative, brute-force search or an approximative, greedy search. Due to the exponential growth of the number of candidate graphs produced, the enumerative search is numerically feasible only in exceptional cases of some low values of *N* and *k*. Therefore, the focus of the numerical investigations is on the approximative search. For this variant we need to specify the filter size $\#_{\mathcal {G}}$ and the filter criteria. Best results were obtained with low values of the algebraic connectivity $\lambda _2$ derived from the normalized Laplacian as filter. We also discuss why the algebraic connectivity $\mu _2$ derived from the standard Laplacian is not likely to be a successful option. According to the filter criteria, the graphs with the lowest $\#_{\mathcal {G}}$ values of $\lambda _2$ are included in the next iteration. If there are less than $\#_{\mathcal {G}}$ graphs, all are taken. Throughout the study we use the filter size $\#_{\mathcal {G}}=500$ as preliminary experiments suggested that such a setting is a good compromise between algorithmic performance and computational effort. The algorithm uses the remeeting time difference $\varDelta ^j \tau _i$ for selecting the vertex from which edges are removed. Further preliminary experiments have shown that a moving difference $\varDelta ^j \tau _i=\tau _i(j)-\tau _i(j-1)$ gives best results and thus is used. This is interesting as a moving difference is a rather rough approximation of the forward difference in Eq. (14). A possible explanation is that Eq. (14) itself is just an approximation of the effect which an edge removal has on the effective population size $N_\textrm{eff}$ indicating a transient amplifier. Thus, the moving difference may possibly describe the effect which step-wise edge removals have on changes of $N_\textrm{eff}$ better than the forward difference. In addition, only $\varDelta ^j \tau _i$ is evaluated to determine from which vertex edges are removed, while Eq. (14) additionally shows contributions of $\varDelta ^q \tau _i$ with $q<j$. Future work should be done to clarify the effect.

Studying the algorithm we have the following performance objectives. First of all, we are interested in the number of transient amplifiers identified. Apart from the actual number of graphs, it is also relevant how many of these graphs are non-isomorphic, which implies they are structurally different. Additionally, our objective is to identify transient amplifiers with small and large $N_\textrm{eff}$ as this implies different amplification properties. However, the algorithm is not explicitly optimizing for large or small $N_\textrm{eff}$ by pruning graphs as has been shown by Möller et al. (2019) using a genetic algorithm (see also an application to finding amplifiers of Bd updating by Allen et al. 2021). Finally, we intend to identify transient amplifiers with different structural properties, as for instance expressed by the mean degree $\bar{k}$. With respect to the behaviour of the algorithm we are mainly interested in how the spectral dynamics generally relates to edge removals and how graph evolutions leading to transient amplifiers differ from evolutions not doing so.

### Regular input graphs on $N=\{11,12\}$ vertices

**Fig. 1:**
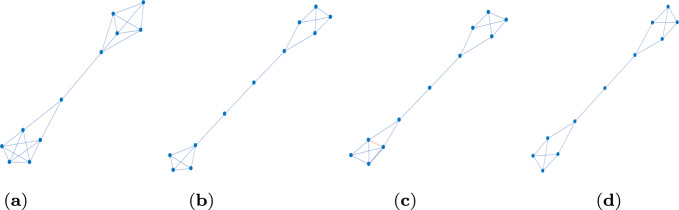
4 non-isomorphic graphs on $N=11$ vertices which are transient amplifiers of death–Birth updating and have maximum degree $\varDelta (\mathcal {G})=4$. **a** $N_\textrm{eff}=11.0008$, $\delta (\mathcal {G})=3$, $\bar{k}=3.8182$, $\lambda _2=0.0567$. **b** $N_\textrm{eff}=11.0128$, $\delta (\mathcal {G})=2$, $\bar{k}=2.9091$, $\lambda _2=0.0399$. **c** $N_\textrm{eff}=11.0056$, $\delta (\mathcal {G})=2$, $\bar{k}=3.0909$, $\lambda _2=0.0451$. **d** $N_\textrm{eff}=11.0952$, $\delta (\mathcal {G})=2$, $\bar{k}=2.9091$, $\lambda _2=0.0481$. This graph can be obtained by removing the edge depicted in red of the graph in Fig. 1c

We start with considering regular graphs on $N=11$ and $N=12$ vertices. For these two graph orders all regular graphs with all occurring degrees have been tested with the numerical resources available in this study. For regular graphs with $N \le 10$ vertices no transient amplifiers of death–Birth updating were found using the method discussed in this paper. Results of Algorithm 1 (approximative, greedy search) for $N=11$ and $k=(4,6,8)$ are given in Fig. 1 and Table 1.

Figure 1 shows 4 non-isomorphic graphs on $N=11$ vertices identified as transient amplifiers of death–Birth updating. The graph in Fig. 1a has the lowest effective population size $N_\textrm{eff}=11.0008$, the graph in Fig. 1b has the highest value $N_\textrm{eff}=11.0128$. All 4 graphs have a maximum degree $\varDelta (\mathcal {G})=4$; the minimum degree is $\delta (\mathcal {G})=3$ for the graph in Fig. 1a and $\delta (\mathcal {G})=2$ for the remaining graphs. Note that the mean degree $\bar{k}$ and the algebraic connectivity $\lambda _2$ scale inversely to the effective population size $N_\textrm{eff}$. All graphs share some structural similarities as they all consist of two cliques of highly connected vertices joined by a bridge, which is a path of one or more edges. Thus, the graphs can be seen as intermediate forms between a dumbbell graph and a barbell graph (Ghosh et al. 2008; Wang et al. 2009), also see the discussion in Sect. 3.4.

**Table 1:** Results of Algorithm 1 (approximative, greedy search) for $N=11$ and $k=(4,6,8)$

*k*	$\mathcal {L}_k$	$\mathcal {A}_k$	$\#_\textrm{tot}$	$\#_\textrm{noniso}$
4	265	5	6	2
6	266	228	937	4
8	6	5	15	1

$\mathcal {L}_k$ is the total number of simple, connected, pairwise nonisomorphic *k*-regular graphs on 11 vertices. $\mathcal {A}_k$ is the proportion of these regular graphs from which transient amplifiers of death–Birth updating are obtained. $\#_\textrm{tot}$ is the total number of transient amplifiers found, $\#_\textrm{noniso}$ is the number of pairwise non-isomorphic transient amplifiers for each *k*. From all graphs with $N=11$ and $k=(4,6,8)$ 4 non-isomorphic are obtained, see Fig. 1

Table 1 gives results about the algorithmic process for $N=11$ and $k=(4,6,8)$. $\mathcal {L}_k(N)$ denotes the total number of simple, connected, pairwise nonisomorphic *k*-regular graphs on *N* vertices. $\mathcal {A}_k(N)$ is the proportion of these regular graphs from which transient amplifiers of death–Birth updating are obtained. A first result is that there are instances of quartic as well as sextic as well as octic regular graphs which can be disturbed into transient amplifiers, but their numbers vary. Whereas for 6-regular graphs $\mathcal {A}_6=228$ graphs out of the $\mathcal {L}_6(11)=266$ and for 8-regular graphs $\mathcal {A}_8=5$ graphs out of the $\mathcal {L}_8(11)=6$ produce transient amplifiers, the percentage of 4-regular graphs is much lower (5 out of 265). Moreover, we know that the result for $k=4$ is not specific for the approximative search with filter size $\#_{\mathcal {G}}=500$ as for the $\mathcal {L}_4=265$ quartic graphs a complete enumeration has been possible and brought exactly the same result. A second finding is that although the total number of transient amplifiers found varies substantially between the graph degrees (there are 937 for $k=6$, but only 6 for $k=4$), the number of non-isomorphic graphs is more stable. Overall, 4 non-isomorphic transient amplifier graphs have been found for $N=11$, see Fig. 1. All 4 graphs are obtained for $k=6$, but the graph in Fig. 1a is also identified for $k=4$ and $k=8$ and the graph in Fig. 1b we also get for $k=4$. In other words, for all degrees of regular graph with $N=11$ used as an input to Algorithm 1, there is a certain convergence toward graph structures having amplification properties.

**Fig. 2:**
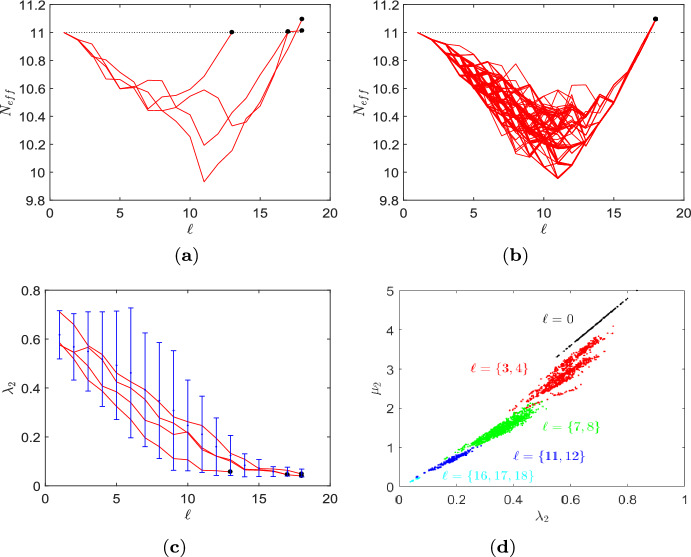
Behaviour of the approximative search of Algorithm 1 for order $N=11$, degree $k=6$. **a** The effective population size $N_\textrm{eff}$ over edge removal repetitions $\ell$ for each of the 4 non-isomorphic transient amplifiers depicted in Fig. 1. The black dots mark the final values $N_\textrm{eff}>11$. **b** $N_\textrm{eff}$ over $\ell$ for the graph with the highest final $N_\textrm{eff}$, Fig. 1b, for all 33 sextic regular graphs which can be disturbed into this transient amplifier. **c** The algebraic connectivity $\lambda _2$ over $\ell$ for all 4 non-isomorphic transient amplifiers according to Fig. 1 and produced by taking 6-regular graphs as input. **d** Scatter plot of algebraic connectivity $\lambda _2$ and $\mu _2$ derived from the normalized and standard Laplacian, respectively, for different edge removal repetitions $\ell$. Black dots $\ell =0$, red dots $\ell =\{3,4\}$, green dots $\ell =\{7,8\}$, blue dots $\ell =\{11,12\}$, cyan dots $\ell =\{16,17,18\}$

Further insight into the algorithmic process can be obtained by analyzing the behavior of some quantities connected to finding transient amplifiers over the run time of the algorithm. Figure 2a shows the effective population size $N_\textrm{eff}$, Eq. (4), over edge removal repetitions $\ell$ for all 4 non-isomorphic transient amplifiers according to Fig. 1 and produced by taking 4 specific 6-regular graphs as input. In other words, each curve in Fig. 2a can be interpreted as a trajectory accounting for $N_\textrm{eff}$ over graphs experiencing repeated edge removals at iterations $\ell$. As for regular graph the equality $N_\textrm{eff}=N$ applies, the curves start at $N_\textrm{eff}=11$, fall to some lower values $10<N_\textrm{eff}<11$, before rising up and ending at specific values $N_\textrm{eff}>11$. These values are marked by black dots for each of the graphs. The graph in Fig. 1a with $N_\textrm{eff}=11.0008$ is obtained for the lowest $\ell$ as it requires the lowest number of edge removals, and thus has the highest mean degree $\bar{k}=3.8182$. The graphs obtained for the highest $\ell$ (shown in Fig. 1b, d) have the lowest mean degree $\bar{k}=2.0991$. The connected black dots of a trajectory between $\ell =17$ and $\ell =18$ indicate that the two transient amplifier graphs differ in just one edge. By removing a single edge (depicted in red) the graph in Fig. 1c can be turned into the graph in Fig. 1d.

Figure 2b illustrates a different aspect of the same process. Here, the effective population size $N_\textrm{eff}$ is shown over edge removal repetitions $\ell$ only for the graph with highest $N_\textrm{eff}=11.0128$, Fig. 1b, but for all initial sextic regular graphs which can be disturbed into this transient amplifier. We obtain that 33 out of the $\mathcal {A}_6= 228$ graphs have this property. Moreover, we get 99 different trajectories along $\ell$ as for the 33 initial 6-regular graphs there are up to 5 different ways edge removal sequences can lead to the same graph. Whereas basically the same shape of the curves can be observed as in Fig. 2a, it is also worth mentioning that about halfway through the process (about $\ell \approx 10$) a rather large range of $N_\textrm{eff}$ can be seen which merges in a steep increase of $N_\textrm{eff}$ before finally reaching $N_\textrm{eff}=11.0128$. This result can be interpreted as starting from the initial regular graphs, after 2 or 3 edge removal repetitions there emerges a substantial structural diversity of graphs by the edge removal process which finally converge to the transient amplifier.

The curves of the effective population size $N_\textrm{eff}$ over the run time of the algorithm expressed by $\ell$, Fig. 2a, b, are typical for all *N* and *k* tested, compare to Figs. 4a and 7a, c, e. These curves suggest that taking $N_\textrm{eff}$ as a filter criteria for searching transient amplifiers most likely is not a viable option. While we aim at high values of $N_\textrm{eff}$ with finally $N_\textrm{eff}>N$, we have transients where subsequent edges removals yield values of $N_\textrm{eff}$ which temporarily are substantially lower. We may interpret the search for transient amplifiers by edge removals from a regular input graph as an optimization problem with an associated fitness landscape. Doing so we would obtain a barrier landscape. Such barrier landscapes are known to be difficult to search as they require valley crossings (Richter and Engelbrecht 2014; van Nimwegen and Crutchfield 2000).

The occurrence of structural diversity of graphs is supported by the results given in Fig. 2c which shows an aspect of spectral dynamics with the behaviour of the algebraic connectivity $\lambda _2$ over $\ell$ for all 4 non-isomorphic transient amplifiers according to Fig. 1 and produced by taking 6-regular graphs as input. Again, the values obtained for the final transient amplifiers are marked by black dots. The blue error bars indicate the range between largest and smallest $\lambda _2$ in the whole ensemble of candidate graphs at iteration $\ell$. We see that the values of $\lambda _2$ leading to transient amplifiers are mostly below the mean algebraic connectivity $\bar{\lambda }_2$ over all candidate graphs, but not the smallest. The values of $\lambda _2$ are mostly falling for $\ell$ getting larger. The interlacing result of normalized Laplacian is $0< \lambda _2(\mathcal {G}-e_{ij}) \le \lambda _3(\mathcal {G})$ which implies that $\lambda _2$ may also increase if an edge is removed. In fact, this can be observed, albeit rarely, for instance at $\ell =9$. However, for most edge removals, $\lambda _2$ is falling or stays constant. Also note that the range of $\lambda _2$ as indicated by the error bars initially increases and takes the largest range at $5 \le \ell \le 10$ before shrinking for the final edge removals prior to eventually obtaining a transient amplifier. The transient amplifiers reached at the end of the process have similar $\lambda _2$ and these values are also taken if the number of edge removals required is smaller as to be seen for the graph in Fig. 1a which occurs for $\ell =11$.

Finally, we analyze how the algebraic connectivity $\lambda _2$ and $\mu _2$ derived from the normalized and the standard Laplacian, respectively, evolve for the edge removal process, see Fig. 2d. The setting is the same as for Fig. 2c, that is for all 4 non-isomorphic transient amplifiers according to Fig. 1 and produced by taking 6-regular graphs as input. Figure 2d shows a scatter plot of $\lambda _2$ and $\mu _2$ over edge removal repetitions $\ell$, where black dots are for $\ell =0$, red dots for $\ell =\{3,4\}$, green dots for $\ell =\{7,8\}$, blue dots for $\ell =\{11,12\}$ and cyan dots for $\ell =\{16,17,18\}$. This means in addition to the edge removals also the relationship between $\lambda _2$ and $\mu _2$ for the sextic regular input graphs is shown (as black dots). As for regular graphs the normalized Laplacian is $\varLambda _{\mathcal {G}}=I-1/k \cdot A$ and the standard Laplacian is $L_{\mathcal {G}}=kI-A$, we have a linear relation $k \lambda _2=\mu _2$ for $\ell =0$, which can be seen as the line of black dots in Fig. 2d. However, for $\ell$ getting larger, edges getting removed and the regularity of the candidate graphs being disturbed, the linear relationship collapses. This is particularly visible for $\ell =\{3,4\}$, see the cloud of red dots in Fig. 2d. This effect gets less profound for $\ell$ getting larger and almost vanishes for $\ell \ge 16$. The result can be interpreted as follows. Particularly in the initial phase of the edge removals both types of algebraic connectivity $\lambda _2$ and $\mu _2$ account for different aspects of the graph structure, and thus might be differently suitable for guiding the search process. The spectrum of the normalized Laplacian capturing geometric and structural properties differently to the spectra of the standard Laplacian or the adjacency matrix has been already noted (Banerjee and Jost 2008, 2009; Banerjee 2012; Gu et al. 2016). Below we come back to this property and discuss more details.

**Fig. 3:**
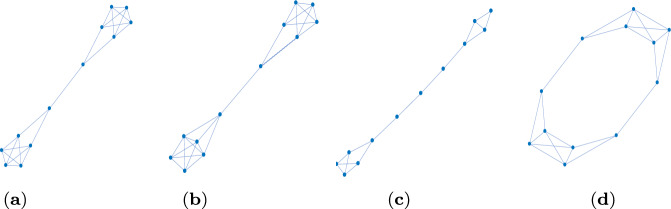
4 examples of 39 non-isomorphic graphs on $N=12$ vertices which are transient amplifiers of death–Birth updating. **a** $N_\textrm{eff}=12.2209$, $\varDelta (\mathcal {G})=4$, $\delta (\mathcal {G})=3$, $\bar{k}=3.8333$, $\lambda _2=0.0455$. **b** $N_\textrm{eff}=12.0390$, $\varDelta (\mathcal {G})=5$, $\delta (\mathcal {G})=3$, $\bar{k}=4.1666$, $\lambda _2=0.0466$. **c** $N_\textrm{eff}=12.0666$, $\varDelta (\mathcal {G})=3$, $\delta (\mathcal {G})=2$, $\bar{k}=2.6666$, $\lambda _2=0.0326$. **d** $N_\textrm{eff}=12.0028$, $\varDelta (\mathcal {G})=4$, $\delta (\mathcal {G})=3$, $\bar{k}=3.6666$, $\lambda _2=0.1134$

The next set of results is for $N=12$, see Fig. 3 and Table 2. We obtain 39 non-isomorphic transient amplifier graphs on $N=12$ vertices. Figure 3 shows 4 examples selected by the largest and smallest effective population size ($N_\textrm{eff}=12.2209$ for the graph in Fig. 3a and $N_\textrm{eff}=12.0028$ for the graph in Fig. 3d), and the largest and smallest mean degree ($\bar{k}=4.1666$ for the graph in Fig. 3b and $\bar{k}=2.6666$ for the graph in Fig. 3c). Again most of the transient amplifier graphs on $N=12$ vertices have structures where two cliques are connected by a single bridge (also compare to the graphs on $N=11$ vertices, see Fig. 1), but there are also 4 graphs where the cliques are connected by two bridges, see an example in Fig. 3d. For this graph structure the algebraic connectivity is $\lambda _2=0.1134$, which is substantially higher than the value of the graphs in Fig. 3a–c. In fact, the mean value for the 35 transient amplifier graphs with just one bridge is $\bar{\lambda }_2=0.0405$, while for the 4 graphs with two bridges it is $\bar{\lambda }_2=0.1088$. The values of the algebraic connectivity $\lambda _2$ are generally known to express some structural properties. Small values of $\lambda _2$ point to large mixing times, bottlenecks, clusters and low conductance (Banerjee and Jost 2008, 2009; Hoffman et al. 2019; Wills and Meyer 2020). Additionally, a low algebraic connectivity indicates path-like graphs which can rather easily be divided into disjointed subgraphs by removing edges or vertices. These are exactly the characteristics we see in the graphs in Fig. 3. The graphs which can be disconnected by removing a single edge (one bridge, Fig. 3a–c) have even lower values of $\lambda _2$ than the graphs where two edges must be removed (two bridges, Fig. 3d).

**Table 2:** Results of Algorithm 1 (approximative, greedy search) for $N=12$ and $k=(3,4,\ldots ,9)$

*k*	$\mathcal {L}_k$	$\mathcal {A}_k$	$\#_\textrm{tot}$	$\#_\textrm{noniso}$	$\bar{\bar{k}}$
3	85	1	1	1	2.8333
4	1.544	226	303	26	3.3141
5	7.848	7.473	43.974	29	3.2931
6	7.849	6.376	63.693	37	3.4595
7	1.547	935	11.989	21	3.6190
8	94	79	557	4	4.0000
9	9	3	23	1	4.1666

$\mathcal {L}_k$ is the total number of simple, connected, pairwise nonisomorphic *k*-regular graphs on 12 vertices. $\mathcal {A}_k$ is the proportion of these regular graphs from which transient amplifiers of death–Birth updating are obtained. $\#_\textrm{tot}$ is the total number of transient amplifiers found, $\#_\textrm{noniso}$ is the number of pairwise non-isomorphic transient amplifiers for each *k*, and $\bar{\bar{k}}$ is the mean degree averaged over these non-isomorphic amplifiers for each *k*. From all graphs with $N=12$ and $k=(3,4,\ldots ,9)$ overall 39 structurally different transient amplifiers are obtained, see Fig. 3 for examples

Table 2 summarizes further results about identifying non-isomorphic transient amplifier graphs on $N=12$ vertices. As for regular graphs on $N=11$ vertices, compare Table 1, for all degrees $k=(3,4,\ldots ,9)$ instances of graphs can be perturbed into transient amplifiers, but again the number of amplifiers differs substantially. Particularly for $k=5$ and $k=6$ a considerable number of *k*-regular input graphs have the property to produce amplifiers. For example, of the 39 structurally different graphs identified, 37 are associated with degree $k=6$. Of the remaining 2, one can be obtained from $k=\{3,4,5\}$ and the other just from $k=\{4,5\}$. Comparing the results for $N=11$ and $N=12$, we see that for a middle range of degrees $k \approx N/2$ the percentage of non-isomorphic (i.e. structurally different) transient amplifiers falls by one order of magnitude. While for $N=11$ and $k=6$ we have $\frac{\#_\textrm{noniso}}{\#_\textrm{tot}}=\frac{4}{937}=4.3 \cdot 10^{-3}$, for $N=12$ there is $\frac{\#_\textrm{noniso}}{\#_\textrm{tot}}=\frac{29}{43.974}=6.6 \cdot 10^{-4}$ for $k=5$ and $\frac{\#_\textrm{noniso}}{\#_\textrm{tot}}=\frac{37}{63.693}=5.8 \cdot 10^{-4}$ for $k=6$. It suggests that for increasing the order from $N=11$ to $N=12$, the algorithmic process constructs roughly 10 times more transient amplifiers which are structurally alike. For several reasons this might appear to be surprising. The total number of pairwise non-isomorphic regular graphs $\mathcal {L}_k$ increases by more than a magnitude, for instance for $k=6$ from $\mathcal {L}_6(11)=266$ for $N=11$ to $\mathcal {L}_6(12)=7.849$ for $N=12$. This would suggest an increased structural diversity of input graphs from which amplifier graphs could emerge. At the same time the number of edges of a regular graph (*kN*/2) increases linearly with *N*, which additionally broadens the possibilities to remove edges and thus for obtaining different structures. At least in principle these possibilities should induce divergence in edge removing trajectories and thus potentially enhance structural diversity in transient amplifiers. However, the results show the contrary. The number of structurally different transient amplifiers increases just about linearly.

There are several possible explanations. A first is that although the search space of possible graph structures increases massively with the graph order *N* rising, transient amplifiers of dB updating are most likely subject to severe structural restrictions which to some extent constrain the feasible search space of candidate graphs, see also the discussion about barbell, dumbbell and other bell-like graphs in Sect. 3.4. Although the algorithmic search discussed in this paper identifies transient amplifiers, they are still relatively rare as compared to the total number of non-isomorphic graphs. Another possibility is that the search process guided by the spectral measure algebraic connectivity $\lambda _2$ actually narrows the search to just a subsection of the overall search space. This certainly is plausible and suggests possible directions for future work on algorithmically identifying transient amplifiers by edge removing procedures, see the discussion in Sect. 4. A third possibility is that the approximative search, and particularly the setting of the filter size $\#_\mathcal {G}$, is responsible for the solely linear increase and a higher number of $\#_\mathcal {G}$ would yield more amplifiers. However, additional experiments with varying $\#_\mathcal {G}$ showed that there is no clear relationship between increasing $\#_\mathcal {G}$ and performance, and higher filter size sometimes even gets worse results. This algorithmic behaviour is a consequences of the iterative process and the fact that small values of $\lambda _2$ point to amplification properties but strictly pursuing only smallest values is not the best option.

**Fig. 4:**
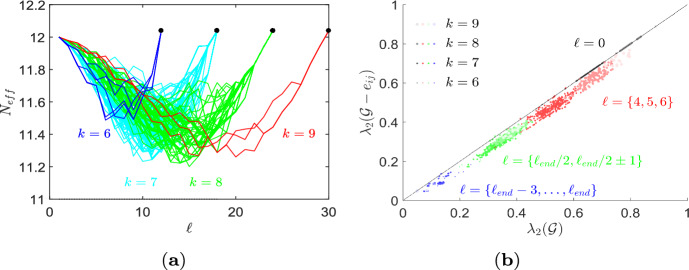
Behaviour of the approximative search of Algorithm 1 for input graphs with order $N=12$ yielding the amplifier with $N_\textrm{eff}=12.0390$ depicted in Fig. 3b and all degrees $k=\{6,7,8,9\}$. **a** The effective population size $N_\textrm{eff}$ over edge removal repetitions $\ell$. **b** Spectral shift in algebraic connectivity $\lambda _2$ induced by removing an edge $e_{ij}$ depicted as scatter plot of $\lambda _2(\mathcal {G}-e_{ij})$ over $\lambda _2(\mathcal {G})$

Figure 4 illustrates further aspects of the algorithmic process. Figure 4a shows the effective population size $N_\textrm{eff}$ over edge removal repetitions $\ell$ for the amplifier depicted in Fig. 3b. This amplifier can be obtained by taking input graphs with 4 different degrees *k*, which is the maximal range of input degrees obtained in the experiments with *k*-regular graphs with $N=12$ and $\#_\mathcal {G}=500$. By taking input graphs with any degree from $k=\{6,7,8,9\}$, we see that for each input degree the final value of $N_\textrm{eff}=12.0390$ is obtained after a specific number of edge removals $\ell =\ell _\textrm{end}$ with $k=6$ needing the smallest $\ell _\textrm{end}$ and $k=9$ needing the largest. The curves for each *k* resemble each other with setting out at $N_\textrm{eff}=12$ and experiencing a prolonged decline to values $N_\textrm{eff}<12$. Afterwards, they spread out to a larger range of $N_\textrm{eff}$ reflecting structural diversity, before the different paths of edge removal trajectories sharply rise and merge before ending at $N_\textrm{eff}=12.0390>12$. The actual amount of edge removals required for different input degrees *k* mainly influences the length of the curves, but not their shape.

These shape similarities point at underlying similarities in the way edges are removed from the input graph. They also becomes noticeable in the graph spectra, see Fig. 4b which shows the spectral shift over edge removals. The spectral shift is depicted as a scatter plot of the algebraic connectivity $\lambda _2(\mathcal {G}-e_{ij})$ over $\lambda _2(\mathcal {G})$ for the candidate graphs $\mathcal {G}$ before and after an edge $e_{ij}$ is deleted. The different colors of the dots indicate different edge removal repetitions $\ell$. The different sizes and lightness of the dots label different input degrees *k*. To compensate for the different $\ell _{end}$ for each $k=\{6,7,8,9\}$, the plot gives the spectral shift for different phases in the edge removing process. The plot in Fig. 4b shows the spectral shift from the regular input graphs experiencing their first edge removal ($\ell =0$, black dots), an initial phase ($\ell =\{4,5,6\}$, red dots), an intermediate phase ($\ell =\{\ell _\textrm{end}/2,\ell _\textrm{end}/2 \pm 1\}$, green dots) and a final phase ($\ell =\{\ell _\textrm{end}-3,\ldots ,\ell _\textrm{end}\}$, blue dots). We particularly see that for the initial edge removal ($\ell =0$, depicted as black dots) almost all value lie on the diagonal $\lambda _2(\mathcal {G}-e_{ij})=\lambda _2(\mathcal {G})$. In other words, there is hardly any spectral shift. This is interesting as the algorithmic search sets $\#_\textrm{lim} \gg k$, and with just *k* possibilities to remove a first edge from a *k*-regular graph, all graphs resulting from the first edge removal are kept as candidate graphs. The filter of the approximative search has no influence on the first step. Therefore, all first edge removals on the trajectory to a transient amplifiers cause no or just a tiny spectral shift. For the number of edge removals $\ell$ increasing this ceases to be the case, although there are still instances of a small or zero spectral shift. There are even rare instances where $\lambda _2(\mathcal {G}-e_{ij})>\lambda _2(\mathcal {G})$, which is known as Braess’s paradox (Eldan et al. 2017). But mostly we have spectral shifts which decrease the algebraic connectivity $\lambda _2$. The majority of values are along a band below the diagonal. The band becomes slightly smaller for $\ell$ getting larger which indicates that the magnitude of the spectral shift lowers. With respect to the different degrees *k* of the input graphs we see that only for the initial phase ($\ell =\{4,5,6\}$, red dots) clearly separable clouds of dots occur while for rising $\ell$ the values are more overlapping. These results support the notion that the edge removing trajectories resemble each other in shape even if their duration differs. We may conclude that the spectral shift along the edge removing process leading to the transient amplifier depicted in Fig. 3b with $N_\textrm{eff}=12.0390$ follows some characteristic patterns. These patterns can similarly be found for the other amplifiers with $N=12$ and analogously for other graph orders *N* as well. The notion of the spectral shift of $\lambda _2$ following characteristic patterns appears to be rather self-evident, giving the fact that the approximative search explicitly selects for graphs with small $\lambda _2$. We next generalize the notion of spectral shifts in three directions, thus studying the spectral dynamics of edge removals. First, we now consider all edge removal repetitions $\ell$ and not only some selected phases, which is expressed as a sum of edge removal $\sum e_{ij}$. Second, all trajectories leading to transient amplifiers are recorded and not just those leading to selected amplifiers, and third, we not only account for the algebraic connectivity $\lambda _2$ but for the whole spectrum $\varLambda _\mathcal {G}=\{\lambda _i(\mathcal {G}-\sum e_{ij})\}$.

**Fig. 5:**
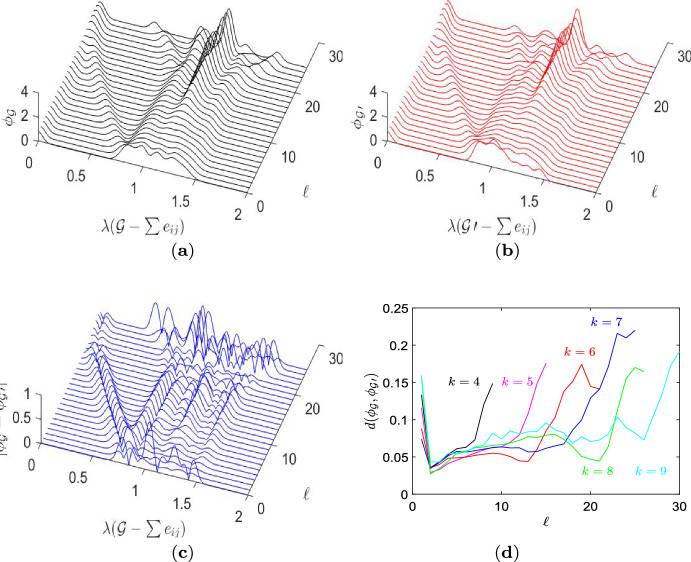
Spectral dynamics of the approximative search of Algorithm 1 for order $N=12$, degree $k=8$. Comparison of the graph set $\mathcal {G}$ (candidate graphs leading to transient amplifiers) with the $\mathcal {G}^\prime$ (candidate graphs not leading to transient amplifiers). **a** The spectral density $\phi _\mathcal {G}$ describing graph evolutions leading to transient amplifiers. **b** The spectral density $\phi _{\mathcal {G}^\prime }$ describing graph evolutions not leading to transient amplifiers. **c** The quantity $|\phi _\mathcal {G}-\phi _{\mathcal {G}^\prime }|$ describing the difference. **d** The spectral distance $d(\mathcal {G},\mathcal {G}^\prime )$, Eq. (16) for any $k=\{3,4,\ldots ,9\}$ for which input graphs on $N=12$ vertices produce transient amplifiers. See Appendix, Figs. 12, 13, and 14, for $\phi _\mathcal {G}$, $\phi _{\mathcal {G}^\prime }$ and $|\phi _\mathcal {G}-\phi _{\mathcal {G}^\prime }|$ of the remaining *k*

Thus, with Fig. 5 we take a broader and more global look at the algorithmic process and examine the dynamics of the whole Laplacian spectra over edge removals from regular input graphs. We consider the spectral density $\phi _\mathcal {G}$ as defined by Eq. (15) which convolves all eigenvalues $\lambda _i$, $i=1,2,\ldots ,N$, with a Gaussian kernel. Thus, the spectral density $\phi _\mathcal {G}$ can be seen as a smoothed curve over the eigenvalue distribution. Furthermore, $\phi _\mathcal {G}$ averages for each $\ell$ over the graph set yielding all transient amplifiers. For instance, for $\ell =0$ we average over all input graphs which finally lead to a transient amplifier. Note that for $\ell =0$ the number of graphs in the graph set is explicitly specified by $\mathcal {A}_k$, see Tables 1 or 2. For $\ell =1$, the graph set comprises of all graphs after the first edge removal which subsequently yield a transient amplifier, and so on for $\ell >1$. Figure 5a shows $\phi _\mathcal {G}$ for input graphs with $k=8$ for all trajectories leading to transient amplifiers. Thus, for $\ell =0$ the graph set consist of $\mathcal {A}_8=79$ out of the $\mathcal {L}_8=94$ input graphs. The results for the other degrees *k* are depicted in the Appendix, see Fig. 12.

There are two interesting features in the spectral dynamics shown in Fig. 5a. The first is that the algebraic connectivity $\lambda _2$ getting progressively smaller and smaller can be seen as a kind of single travelling peak setting out at $\lambda (\mathcal {G}-\sum e_{ij}) \approx 0.9$ for $\ell =0$ and ending at $\lambda (\mathcal {G}-\sum e_{ij}) \approx 0$ for $\ell =25$. Over all graphs the decrease in $\lambda _2$ caused by repeated edge removals is narrowly bounded and almost continuous. This is in contrast to random edge removals which do not exhibit such a behavior. The second important feature is a kind of standing peak at $\lambda (\mathcal {G}-\sum e_{ij}) \approx 1.1$ which becomes prominent at $\ell \approx 10$ and continuously increases thereafter for $\ell \le 25$. Such an increase in $\phi _\mathcal {G}$ indicates a multiplicity of eigenvalues which additionally points at doubling of motifs in the graph (Mehatari and Banerjee 2015). These results can be contrasted with the spectral density of graph evolutions which do not lead to transient amplifiers. Figure 5b shows the spectral density $\phi _{\mathcal {G}^\prime }$ of the graph set $\mathcal {G\prime }$ over $\ell$ and $\lambda ({\mathcal {G}^\prime }-\sum e_{ij})$. The graph set ${\mathcal {G}^\prime }$ consists of all candidate graphs produced on the edge removing trajectory which are not finally leading to a transient amplifier. Thus, ${\mathcal {G}^\prime }$ can be seen as complementary to $\mathcal {G}$. For instance, for $\ell =0$ is comprises of the remaining $\mathcal {L}_k-\mathcal {A}_k$ input graphs, and so on. Comparing the spectral density $\phi _\mathcal {G}$ of the graph set leading to transient amplifiers, Fig. 5a, with $\phi _{\mathcal {G}^\prime }$ not leading to transient amplifiers, general similarities can be noted. Also in Fig. 5b we see the travelling peak indicating decreasing $\lambda _2$ over $\ell$ and the standing peak indicating increasing eigenvalue multiplicity. However, the difference $|\phi _\mathcal {G}-\phi _{\mathcal {G}^\prime }|$, see Fig. 5c, also reveals significant differences in the graph sets.

A first is the difference $|\phi _\mathcal {G}-\phi _{\mathcal {G}^\prime }|$ showing that the travelling peak has a kind of notch which makes the peak appear split and twofold. The geometrical interpretation is that the travelling peak of $\phi _\mathcal {G}$ is more narrow than the one of $\phi _{\mathcal {G}^\prime }$. In other words, the range of progressively decreasing values of $\lambda _2$ is smaller for graphs evolving towards transient amplifiers than for graphs which do not lead to amplifiers. A second is that while in the initial and intermediate phase of edge removals the differences remain within a certain range of $\lambda (\mathcal {G}-\sum e_{ij})$, they spread out in the final phase, particularly for $\ell >20$. This means that in the final phase of edge removals the $\lambda _i$ for graphs evolving towards transient amplifiers are more dispersed than those for graphs not doing so. This most likely indicates that on average graphs evolving towards transient amplifiers build up characteristic structural features which entail certain values and multiplicities in $\lambda _i$. These features become visible in the spectral density $\phi _\mathcal {G}$. Candidate graphs which do not evolve towards amplification properties do not specifically possess these features. In the spectral density $\phi _{\mathcal {G}^\prime }$ the resulting variety of structural features cancels off, leading to differences as compared to $\phi _\mathcal {G}$. For the other degrees *k*, we find similar characteristics, see Figs. 12, 13, and 14 in the Appendix. An exception is $k=3$ for which there is only a single edge removal from input graph to transient amplifier and not the same characteristic curves. However, to some extend for $k=4$ and clearly for $k>4$ the features described become visible.

**Table 3:** Results of Algorithm 1 (approximative, greedy search) for $N=\{14,20,26\}$ and $k=(11,17,23)$

*N*	*k*	$\mathcal {L}_k(N)$	$\mathcal {A}_k(N)$	$\#_\textrm{tot}$	$\#_\textrm{noniso}$	$\bar{\bar{k}}$
14	11	13	4	1.597	9	5.2540
20	17	49	2	4.212	43	8.4163
26	23	130	13	58.355	55	11.6154

$\mathcal {L}_k(N)$ is the total number of simple, connected, pairwise nonisomorphic *k*-regular graphs on *N* vertices. $\mathcal {A}_k(N)$ is the proportion of these regular graphs from which transient amplifiers of death–Birth updating are obtained. $\#_\textrm{tot}$ is the total number of transient amplifiers found, $\#_\textrm{noniso}$ is the number of pairwise non-isomorphic transient amplifiers for each *k*, and $\bar{\bar{k}}$ is the mean degree averaged over these non-isomorphic amplifiers for each *k*

For an overall comparison between the spectral densities, the spectral distance $d(\mathcal {G},{\mathcal {G}^\prime })$, Eq. (16) can be used. Figure 5d shows this quantity for any $k=\{4,\ldots ,9\}$ for which input graphs on $N=12$ vertices produce transient amplifiers. The degree $k=3$ is omitted as there is only a single edge removal from input graph to transient amplifier and thus no meaningful comparison over $\ell$. We see that although the number of required edge removals varies for different *k*, the spectral distance starts at large values for $\ell =0$, before dropping for a certain amount of time but increasing again for the graph evolution about to finish towards transient amplifiers. In other word, at the beginning and at the end of the edge removal process, the graph set connected with amplifiers and the graph set not connected with amplifiers have clear differences in their normalized Laplacian spectra.

**Fig. 6:**
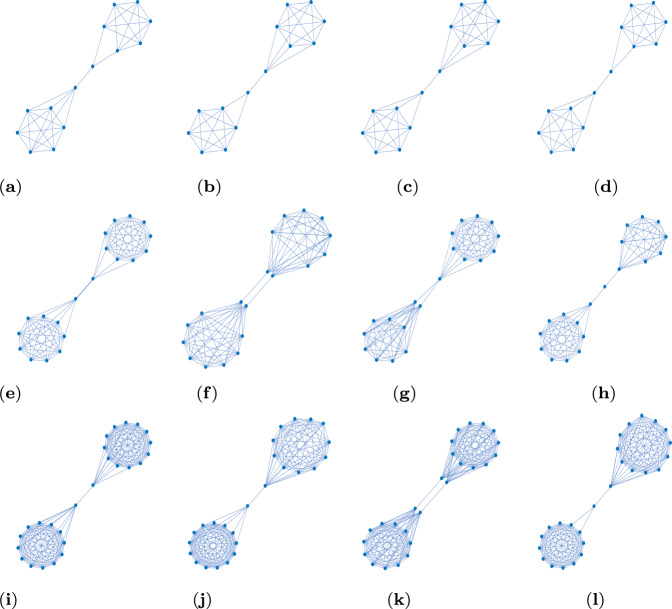
Examples of non-isomorphic graphs on *N* vertices which are transient amplifiers of death–Birth updating. **a**–**d**: $N=14$, which have maximum degree $\varDelta (\mathcal {G})=6$. **e**–**h**: $N=20$, which have maximum degree $\varDelta (\mathcal {G})=9$. **i**–**l**: $N=26$, which have maximum degree $\varDelta (\mathcal {G})=12$ except **j**, which has $\varDelta (\mathcal {G})=13$. The values of the effective population size $N_\textrm{eff}$, the minimum degree $\delta (\mathcal {G})$, the mean degree $\bar{k}$ and the algebraic connectivity $\lambda _2$ are

**Table:** 

	(a)	(b)	(c)	(d)	(e)	(f)	(g)	(h)
$N_\textrm{eff}$	14.0986	14.0073	14.0247	14.0533	20.1978	20.0013	20.0021	20.0831
$\delta (\mathcal {G})$	3	3	4	3	5	8	8	2
$\bar{k}$	5.1429	5.1429	5.4286	5.1429	8.2000	8.9000	8.8000	7.9000
$\lambda _2$	0.0331	0.0329	0.0350	0.0313	0.0172	0.0383	0.0330	0.0113

	(i)	(j)	(k)	(l)				
$N_\textrm{eff}$	26.1747	26.0019	26.0092	26.0170				
$\delta (\mathcal {G})$	9	6	11	3				
$\bar{k}$	11.5385	11.3077	11.9231	11.2308				
$\lambda _2$	0.0110	0.0108	0.0227	0.0086				

### Regular input graphs on $N=\{14,20,26\}$ vertices

As shown in the previous section, for regular graphs on $N=11$ and $N=12$ vertices it has been possible with the available computational resources to treat inputs from all structurally different graphs with all existing degrees. For $N\ge 14$ this has not been feasible due to the massive growth of the number of potential input graphs $\mathcal {L}_k(N)$ (Meringer 1999; Richter 2021; WolframMathWorld 2022). For nevertheless studying a closed set of graphs covering a complete structural range, we next consider inputs on $N=\{14,20,26\}$ vertices with degree $k=N-3$. Thus, we have a complete structural variety for the given *N* and *k*, while the total number of input graphs remains computationally manageable, see Table 3 and Fig. 6.

With increasing order of the considered input graphs also a rising number of transient amplifiers has been identified. Figure 6 gives 4 examples each for $N=\{14,20,26\}$ out of the $\#_\textrm{noniso}=\{9,43,55\}$ non-isomorphic amplifiers according to Table 3. The examples are again selected by the largest and smallest values of the effective population size $N_\textrm{eff}$ and the mean degree $\bar{k}$. In addition, the maximum and minimum degree, $\varDelta (\mathcal {G})$ and $\delta (\mathcal {G})$, as well as the algebraic connectivity $\lambda _2$ are given. By comparing the obtained graphs we once more observe characteristic structural features favoring amplification. We find again exclusively graphs consisting of two highly connected cliques. They are mostly joined by a single bridge of one or two edges, but there are also rare instances with two bridges. Next to these structural similarities there are also differences for a varying number of vertices. For the order of input graphs going up also the maximum, minimum and mean degree ($\varDelta (\mathcal {G})$, $\delta (\mathcal {G})$, and $\bar{k}$, respectively) increase with an approximately linear ratio. For transient amplifiers with fixed order *N* we see no or very little variance in the maximum degree $\varDelta (\mathcal {G})$, which also applies for the mean degree $\bar{k}$. The largest variance can be found for the minimum degree $\delta (\mathcal {G})$, which can be as low as $\delta (\mathcal {G})=2$ for bridges with two or more edges, but also as high as $\delta (\mathcal {G})=\varDelta (\mathcal {G})-1$ for amplifiers with two bridges, see for instance Fig. 6f, k. Similarly to the results for $N=12$, the algebraic connectivity $\lambda _2$ of transient amplifiers has very small values, while the examples with two bridges have largest.

**Fig. 7:**
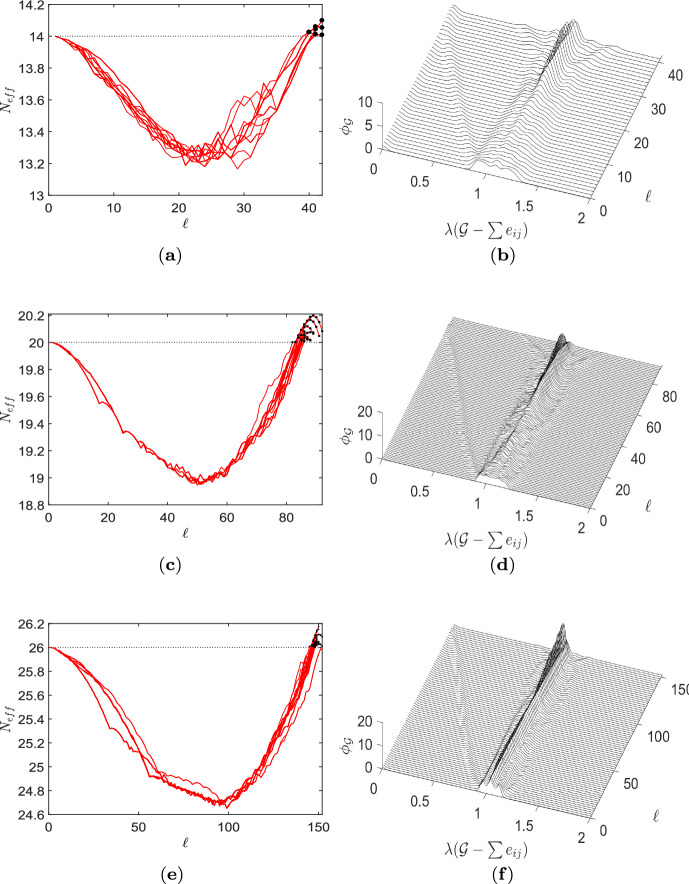
Behaviour of the approximative search of Algorithm 1 for $N=\{14,20,26\}$ and $k=\{11,17,23\}$. The effective population size $N_\textrm{eff}$ and the spectral density $\phi _\mathcal {G}$ describing graph evolutions leading to transient amplifiers over edge removal repetitions $\ell$. **a**,**b** $N=14$, $k=11$. **c**, **d** $N=20$, $k=17$. **e**, **f** $N=26$, $k=23$. See Appendix, Fig. 15, for the spectral density $\phi _{\mathcal {G}^\prime }$ and the difference $|\phi _\mathcal {G}-\phi _{\mathcal {G}^\prime }|$

Figure 7a, c, e gives the behavior of the effective population size $N_\textrm{eff}$ over edge removal repetitions $\ell$ for $N=\{14,20,26\}$. The results are generally similar to $N=12$, compare to Fig. 4a. Though, for $N=20$ and $N=26$ we frequently find graph trajectories with consecutive transient amplifiers. This means for a certain $\ell$ we have a transient amplifier graph and by removing an edge from this graph, we get another transient amplifier. The effective population size $N_\textrm{eff}$ may vary for consecutive transient amplifiers, and we find successively increasing values as well as a parabolic succession. Figure 7b, d, f shows the spectral dynamics expressed by spectral densities $\phi _\mathcal {G}$. Again there is a general similarity to $N=12$, compare to Fig. 5a. Particularly, the two geometrical features already discusses, the travelling peak of $\lambda _2$ progressively getting smaller and the standing peak indicating eigenvalue multiplicity can be found in almost the same manner. Thus, it can be concluded that they are features independent of the considered *N* and *k*. For the spectral density $\phi _{\mathcal {G}^\prime }$ and the difference $|\phi _\mathcal {G}-\phi _{\mathcal {G}^\prime }|$, see Appendix, Fig. 15.

**Fig. 8:**
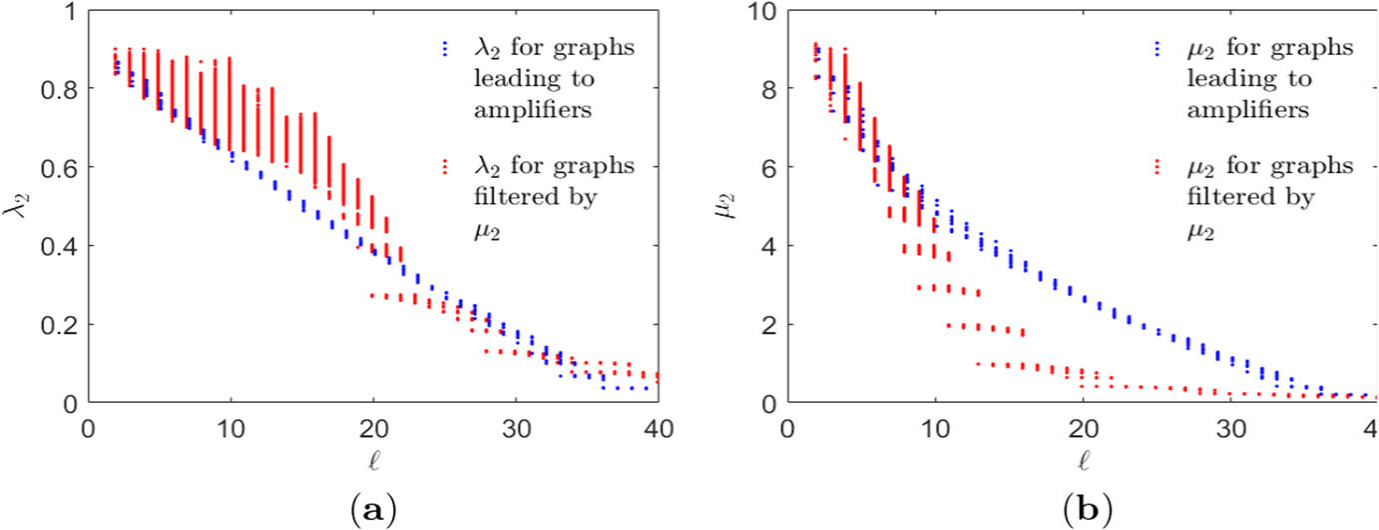
Spectral dynamics of guided edge removals. Comparing between graph evolutions leading or not leading to transient amplifiers evaluated and filtered by spectral graph measures. **a** Algebraic connectivity $\lambda _2$ derived from the normalized Laplacian. **b** Algebraic connectivity $\mu _2$ derived from the standard Laplacian

The transient amplifiers discussed up to now have been identified using as filter the algebraic connectivity $\lambda _2$ derived from the normalized Laplacian. A result worth mentioning is that contrary to using $\lambda _2$ as filter, taking the algebraic connectivity $\mu _2$ derived from the standard Laplacian does not yield amplifiers, at least not for the tested input graphs on $N=\{11,12,14,20,26\}$ vertices and filter sizes from $\#_\mathcal {G}=500$ up to $\#_\mathcal {G}=2.500$. In the discussion about using input graphs on $N=11$ vertices and degree $k=6$, it has been observed that the quantities $\lambda _2$ and $\mu _2$ behave differently for edges being removed from a regular graph, see Fig. 2d. This is the case for all *N* and *k* tested. Using the example $N=14$ and $k=11$ this behaviour is now analysed by their spectral dynamics. It is furthermore argued that such an analysis offers a possible explanation as to why $\lambda _2$ as filter leads to amplifiers while $\mu _2$ does not.

We compare for subsequent edge removal repetitions $\ell$ how graph evolutions leading to transient amplifiers guided by low values of $\lambda _2$ would be evaluated if the filter were using low values of $\mu _2$. The setup of the analysis is this. We take a single input graph from the pool of input graphs finally leading to transient amplifiers. The results given in Fig. 8 are for a graph with $N=14$ and $k=11$ which yields in total 530 amplifiers of which 9 are pairwise non-isomorphic. For other input graphs, also with other *N*, similar results have been obtained. Using this input graph we track the values of $\lambda _2$ and $\mu _2$ for these 530 trajectories leading to amplifiers in the process guided by $\lambda _2$, see the blue dots in Fig. 8a, b. Then, we rerun the edge removal process taking the same input graph and the graphs on the trajectory towards transient amplifiers, but filter and select for each $\ell$ according to $\mu _2$. In other words, we track the values of $\lambda _2$ and $\mu _2$ for the graph evolution leading to amplifiers for each consecutive $\ell$ as if the graphs were to be evaluated and filtered by $\mu _2$, see the red dots in Fig. 8a, b. The results show that for $\lambda _2$, see Fig. 8a, the values of graphs leading to amplifiers are mostly below the values for graphs that would have been taken if they were filtered by $\mu _2$. As the filter selects for small values, graphs leading to amplifiers actually remain in the pool of candidate graphs. There is an interval in edge removals $20<\ell <30$ where the values of $\lambda _2$ for graphs selected according to $\mu _2$ are lower than those on the trajectory towards amplifiers, but if the filter size is large enough this does not lead to an exclusion of candidate graphs needed to finally obtain amplifiers.

If we look at the spectral dynamics from the perspective of $\mu _2$, we get different results, see Fig. 8b. Here the values of $\mu _2$ leading to transient amplifiers are mostly above the values of those filtered by $\mu _2$, particularly for $\ell >10$. Thus, as the filter selects for small values of $\mu _2$ the graphs which would have led to amplifiers are gradually sorted out of the pool of candidate graphs and thus no transient amplifiers are identified. It is quite possible that using $\mu _2$ as filter would lead to amplifiers if the filter size $\#_\mathcal {G}$ is larger than some threshold. However, tests with filter sizes up to $\#_\mathcal {G}=2.500$ have brought no results.

### Barbell, dumbbell and other bell-like graphs

**Fig. 9:**
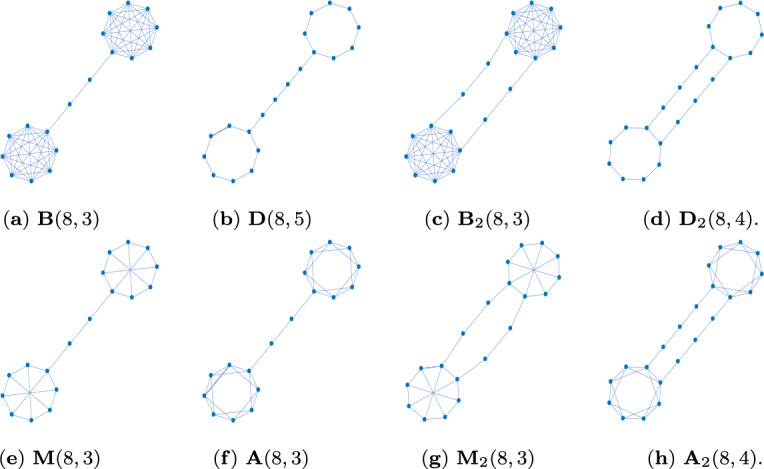
Barbell, dumbbell and other bell-like graphs. The Barbell graphs $\textbf{B}(a,b)$, dumbbell graphs $\textbf{D}(a,b)$, Möbius-ladder-bell graphs $\textbf{M}(a,b)$ and antiprism-bell graphs $\textbf{A}(a,b)$ consisting of two complete graph (or two circle graph, or two Möbius-ladder graphs, or two antiprism graphs) with *a* vertices each and a bridge with *b* edges. The graphs $\textbf{B}_2(a,b)$, $\textbf{D}_2(a,b)$, $\textbf{M}_2(a,b)$ and $\textbf{A}_2(a,b)$ have two bridges

Barbell and dumbbell graphs are two families of graphs with a standardized structure (Ghosh et al. 2008; Wang et al. 2009). Barbell graphs $\textbf{B}(a,b)$ consist of two complete graphs with *a* vertices each which are connected by a bridge with *b* edges, while dumbbell graphs $\textbf{D}(a,b)$ consist of two circles with *a* vertices each which are also connected by a bridge with *b* edges. According to such a definition a $\textbf{B}(a,b)$ barbell graph as well as a $\textbf{D}(a,b)$ dumbbell graph has $N=2a+b-1$ vertices, see the barbell graph $\textbf{B}(8,3)$ in Fig. 9a and the dumbbell graph $\textbf{D}(8,5)$ in Fig. 9b. In addition, we discuss two more families of bell-like graphs, which use two circulant graphs as building blocks, Möbius ladder graphs and antiprism graphs. A Möbius ladder graph consists of a cycle graph with *a* vertices and additional edges connecting opposite pairs of vertices as rungs, while an antiprism graph involves an antiprism as its skeleton (Read and Wilson 1998). We call them Möbius-ladder-bell graphs $\textbf{M}(a,b)$ and antiprism-bell graphs $\textbf{A}(a,b)$. They consists of two Möbius ladder graphs (or two antiprism graphs) with *a* vertices each which are also connected by a bridge with *b* edges, see the Möbius-ladder-bell $\textbf{M}(8,3)$ in Fig. 9e and the antiprism-bell graph $\textbf{A}(8,3)$ in Fig. 9f. In view of the findings that most, if not all, amplifier graphs identified using the algorithmic framework discussed in the previous sections resemble barbell and dumbbell graphs in some way or another, we next discuss amplification properties of these graphs.

**Fig. 10:**
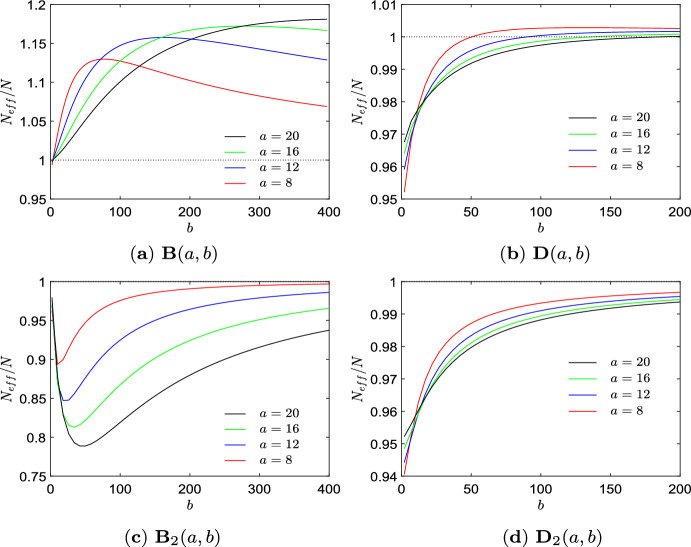
The quantity $N_\textrm{eff}/N$ for barbell graphs $\textbf{B}(a,b)$ and $\textbf{B}_2(a,b)$ as well as dumbbell graphs $\textbf{D}(a,b)$ and $\textbf{D}_2(a,b)$ for different *a* over *b*. The variable *a* is the number of vertices in each of the two complete (or circle) graphs, *b* is the number of edges in the bridge or the two bridges, see also Fig. 9. Values of $N_\textrm{eff}/N>1$ indicate transient amplification properties

**Fig. 11:**
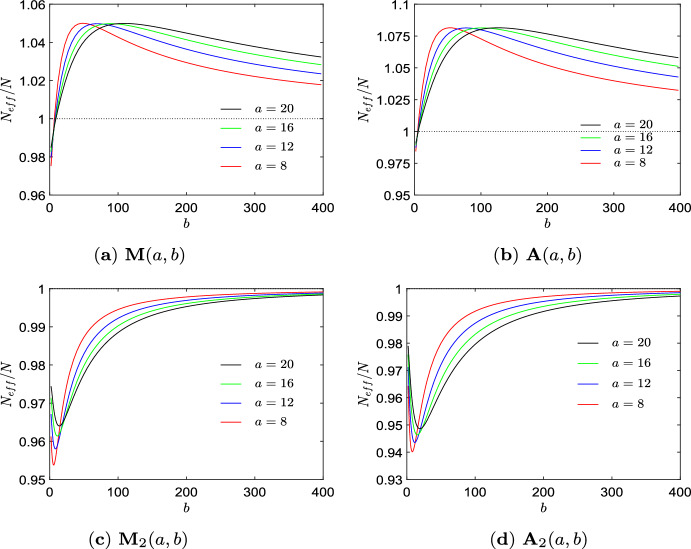
The quantity $N_\textrm{eff}/N$ for Möbius-ladder-bell graphs $\textbf{M}(a,b)$ and $\textbf{M}_2(a,b)$ as well as for antiprism-bell graphs $\textbf{A}(a,b)$ and $\textbf{A}_2(a,b)$ for different *a* over *b*. The variable *a* is the number of vertices in each of the two Möbius-ladder (or antiprims) graphs, *b* is the number of edges in the bridge or the two bridges, see also Fig. 9. Values of $N_\textrm{eff}/N>1$ indicate transient amplification properties

Figure 10a gives the quantity $N_\textrm{eff}/N$ for barbell graphs $\textbf{B}(a,b)$ with $a=\{8,12,16,20\}$ and $1\le b \le 400$. A ratio $N_\textrm{eff}/N>1$ indicates transient amplification properties. We see that for all *a* and $b>3$ transient amplifiers exist. The ratio $N_\textrm{eff}/N$ increases with rising *b* (and by $N=2a+b-1$ with rising *N*) for a certain interval in *b*, before reaching a maximum and then slowly falling and finally converging to a value $N_\textrm{eff}/N>1$. The larger the value *a* is (indicating the number of vertices in the two complete subgraphs) the higher is the maximum $N_\textrm{eff}/N$ itself and the larger the associated number of bridges *b*. The smallest barbell graph with amplification properties is $\textbf{B}(4,5)$, which has $N=12$. For dumbbell graph the results are qualitatively similar, but some details differ, see Fig. 10b for the same values of *a* and $1\le b \le 200$. Also for dumbbell graphs amplification properties can be found, but the ratio $N_\textrm{eff}/N$ is much smaller than for barbell graphs and also the number of bridges (and thus the order of the graph) needed is higher. The smallest dumbbell graph with amplification properties is $\textbf{D}(3,12)$, which has $N=17$. Generally speaking, amplification properties of barbell and dumbbell graphs are universal and for other values of *a* characterising the number of vertices in the bell-like clique, similar results are obtained. These results are confirmed by Möbius-ladder-bell graphs $\textbf{M}(a,b)$ and antiprism-bell graphs $\textbf{A}(a,b)$, see Fig. 11a, b. The smallest Möbius-ladder-bell graph and the smallest antiprism-bell graph which are transient amplifiers are $\textbf{M}(4,5)$ and $\textbf{A}(4,5)$, which both have (as the smallest barbell graph) $N=12$. Generally speaking, the ratio $N_\textrm{eff}/N$ is between barbell and dumbbell graphs (and for Möbius-ladder-bell graphs smaller than for antiprism-bell graphs) which suggests the speculation that the higher the degree of the bell-like clique (for a complete graph $k=N-1$, for the antiprism graph $k=4$, for the Möbius ladder graph $k=3$ and the cycle graph $k=2$) the higher the potential amplification.

As some of the algorithmically identified transient amplifiers have two bridges (see for instance, Fig. 3d or Fig. 6f, k), we finally study amplification properties of barbell, dumbbell and other bell-like graphs with two bridges. Therefore, we define two bridge barbell graphs $\textbf{B}_2(a,b)$ as two complete graphs with *a* vertices each connected by two bridges of *b* edges each. As in a complete graph each vertex is connected to all other vertices (except itself) it makes no difference which two vertices serve as bridgeheads. A dumbbell graph $\textbf{D}_2(a,b)$, a Möbius-ladder-bell graph $\textbf{M}_2(a,b)$ and a antiprism-bell graphs $\textbf{A}_2(a,b)$ with two bridge each is defined likewise, but here the location matters where the bridges branch off. We define that the two bridgeheads on each side are directly connected, see Fig. 9c, d, g, h for the examples of $\textbf{B}_2(8,3)$, $\textbf{D}_2(8,4)$, $\textbf{M}_2(8,3)$ and $\textbf{A}_2(8,4)$. With respect to transient amplification, we see that most likely neither $\textbf{B}_2(a,b)$ nor $\textbf{D}_2(a,b)$, $\textbf{M}_2(a,b)$ or $\textbf{A}_2(a,b)$ have this properties, see Figs. 10c, d and 11c, d which give the ratio $N_\textrm{eff}/N$ for different *a* over *b*. We observe that the curves are always below $N_\textrm{eff}/N=1$, become lower for *a* increasing with $N_\textrm{eff}/N \rightarrow 1$ from below for *b* getting large. Also, tests with other *a* and *b* have not revealed amplification. Eventually, minor modification in the bell-like graphs were introduced, for instance deleting the edge between the bridgeheads of the $\textbf{B}_2(a,b)$ barbell graphs or varying the edge distance between the bridgeheads of the $\textbf{D}_2(a,b)$, $\textbf{M}_2(a,b)$ or $\textbf{A}_2(a,b)$ graphs, or having different amounts of vertices in the two bell-like cliques, or connecting the bridge to two bridgeheads. However, also these graphs have not shown amplification properties. It remains to be observed that although transient amplifiers with two bridges have been found, for instance, Fig. 3d or Fig. 6f, k, there should be more subtle rules as to how barbell, dumbbell and other bell-like graphs must be modified to possess this property. For instance, the examples of two bridge transient amplifiers have bell-like cliques which taken as subgraphs are close to regularity, but with a few edges removed from a regular graph. However, this property, if widespread, makes it not very likely that regular graphs are directly usable as building blocks for two bridge transient amplifiers. This may be a topic for future work.

## Discussion

### Identifying transient amplifiers

In the previous section, results about using an iterative algorithmic process for identifying transient amplifier for dB updating have been presented. We next discuss some implications of the results obtained. The algorithm has been tested for all regular graphs on $N=\{11,12\}$ vertices and all degrees, and all regular graphs on $N=\{14,20,26\}$ vertices and degree $k=N-3$. It has been shown that although transient amplifiers for dB updating are rather rare, a substantial number of instances has been identified for all tested graph orders. For $N=11$ and $N=12$, most structurally different transient amplifiers are obtained for middle range *k*, that is $k \approx N/2$. It seems to be reasonable to assume that this also applies to $N=\{14,20,26\}$ and the amplifiers obtained for $k=N-3$ are just a small subset of all amplifiers. Unfortunately, a direct test of this assumption was not possible with the available numerical resources due to the massive growth in structurally different regular input graphs (for instance, there are $\mathcal {L}_7(14)=21.609.301$ regular graphs for degree $k=7$ and order $N=14$, $\mathcal {L}_{10}(20)$ and $\mathcal {L}_{13}(26)$ are still not exactly known).

Furthermore, all amplifier graphs share certain structural characteristics. They are graphs consisting of two cliques of highly (frequently completely) connected vertices, which are joined by a bridge of one or more edges. Occasionally, structures with two bridges connecting the cliques have amplification properties. Moreover, these structures resemble those of barbell, dumbbell and other bell-like graphs, which themselves have amplification properties, see Sect. 3.4. Considering the space of all structurally different graphs with a given order *N*, these structures are rather special and consequently rare. This is in agreement with a previous work (Richter 2021) studying the structural and spectral properties connected with removing a single edges from cubic (and quartic) regular graphs up to an order of $N=22$ (and $N=16$). Also these results showed that transient amplifiers for dB updating exist for all *N* tested, are really rare and have certain graph structures. Extending these results, in this study we have been interested in the transition process from a regular graph to a transient amplifier over multiple edge removals. Thus, we obtained a larger variety of transient amplifiers with a stronger perturbation to the regularity of the input graphs. Nevertheless, also this larger variety is subject to similar structural restrictions. One way of accounting for these restrictions is the degree distribution of graphs $\mathcal {G}$ expressed by the maximum degree $\varDelta (\mathcal {G})$, the minimum degree $\delta (\mathcal {G})$ and the mean degree $\bar{k}$. If we compare over varying order *N*, we see that generally the maximum degree $\varDelta (\mathcal {G})$ is bounded by $\frac{1}{3}N< \varDelta (\mathcal {G}) < \frac{1}{2}N$, while the mean degree $\bar{k}$ is restricted to $\frac{4}{5}\varDelta (\mathcal {G})<\bar{k}<\varDelta (\mathcal {G})$.

Only for the minimum degree $\delta (\mathcal {G})$ we find a rather large variety, which can be as low as $\delta (\mathcal {G})=2$ for transient amplifiers with bridges of two and more edges, or as high as $\delta (\mathcal {G})=\varDelta (\mathcal {G})-1$ for some amplifiers with two bridges. In other words, transient amplifiers seems to have an upper and lower bound of the maximal and the mean degree. Such a distribution of the mean degree $\bar{k}$ differs from random graphs, for instance Erdös–Rényi or Barabási–Albert graphs, which have a binomial and power-law distribution with a much larger spread. Furthermore, this means that the degree *k* of the regular input graph plays a role in what structure a transient amplifier actually has only insofar as it bounds the maximum degree $\varDelta (\mathcal {G})$. This is particularly visible for input graphs on $N=12$ vertices where for all degrees $k=\{3,4,\ldots ,9\}$ transient amplifiers have been identified, see Table 2. If we compare over varying input degrees *k*, we see that the mean degree $\bar{k}$ slightly increases with increasing *k* but the transient amplifiers remain in a rather small range of $\bar{k}$ ($2.6666 \le \bar{k} \le 4.1666$). In other words, the input degree *k* does not matter very much. If the input degree *k* is much larger than the upper bound of the range then just more edges need to be removed before a transient amplifiers appears. Thus, a main result of this study is that many graph structures resembling barbell, dumbbell and other bell-like graphs with two cliques of highly connected vertices joined by a bridge are transient amplifier of dB updating. These structures expand the collection of graph structures already known to have this property, which are known as fans, separated hubs and stars of islands (Allen et al. 2020). They also complement graph structures known as amplifiers of Bd updating and called lollipop, balloon, balloon-star graphs (Allen et al. 2021; Möller et al. 2019).

An interesting question is why the iterative algorithmic process does head for graph structures resembling barbell, dumbbell and other bell-like graphs but not for structures similar to fans, separated hubs or stars of islands. A main reason most likely is that the approximative search using as a filter small values of the algebraic connectivity $\lambda _2$ particularly promotes such structures. The value $\lambda _2=0$ means a disconnected graph, and low values of $\lambda _2$ imply closeness to disconnection, but also bottlenecks, clusters, low conductance and path-like graphs which can rather easily be divided into disjointed subgraphs by removing edges or vertices (Banerjee and Jost 2008, 2009; Hoffman et al. 2019; Wills and Meyer 2020). Fans, separated hubs or stars of islands are structurally further away from being close to disconnection than bell-like graphs. It could be an topic of future work if a filter using different spectral or other graph measures apart from (or in addition to) the algebraic connectivity would be suitable to identify also these structures.

### Spectral dynamics of guided edge removals

In this study we are equally interested in the performance and the behavior of the iterative process for identifying transient amplifier for dB updating. While Sect. 4.1 mainly focused on algorithmic performance, we next discuss some aspects of algorithmic behaviour. A main tool in analyzing the algorithmic behaviour is the spectral dynamics of guided edge removals from regular input graphs. The search process is guided by two quantities derived from the graph, the largest remeeting time $\tau _i$ and the algebraic connectivity $\lambda _2$ of the normalized Laplacian. Both quantities guide the search on different levels. The largest remeeting time $\tau _i$ is suitable to compare vertices and determines from which vertex an edge should be removed. It is not suitable for comparing graphs, but the algebraic connectivity $\lambda _2$ is. It determines for the approximative search which graphs remain in the pool of candidate graphs. The decision to use the quantity $\lambda _2$ as a filter for candidate graphs is itself a result of preliminary analysis and previous work. On the one hand, previous results showed that one edge removals yielding transient amplifiers are connected with low $\lambda _2$ (Richter 2021). Moreover, there are applications of graph breeding and graph pruning algorithms in network science which successfully used spectral properties, particularly algebraic connectivity, for guiding the search process (Chan and Akoglu 2006; Ghosh and Boyd 2006; Ghosh et al. 2008; Li et al. 2018; Shine and Kempe 2019; Sydney et al. 2013). Finally, a preliminary analysis revealed that local graph measures such as betweenness or closeness centrality and degree distribution, but also motive and cycle count somehow correlate to graph evolutions leading to transient amplifiers, but are generally not promising as filter criteria. The lack of usefulness of another global graph measure, the algebraic connectivity $\mu _2$ associated with the standard Laplacian, has been discussed in Sect. 3.3, see also Fig. 8.

Spectral dynamics generally refers to changes in the graph spectra over graph manipulations (Chen and Zhang 2017; Zhang et al. 2009). We here consider the graph manipulations to be repeated edge removals. In Sects. 3.2 and 3.3 several instances are given of how the algebraic connectivity $\lambda _2$ as well as the smoothed spectral density $\phi _\mathcal {G}$ changes if we remove edges from a regular input graph and either obtain a transient amplifiers in the end, or not. These results demonstrate that the spectral dynamics towards transient amplifiers subtly differs from the spectral dynamics of graph evolutions not doing so. This is particularly visible if we consider the spectral dynamics of the smoothed spectral density $\phi _\mathcal {G}$ representing the whole normalized Laplacian and focus on the initial and the final phase of the edge removals. The spectral dynamics towards amplifiers is also substantially different from graph evolutions which are not guided, for instance random graphs and random edge removals. Thus, the results of this paper also expand the applications of spectral analysis of evolutionary graphs (Allen et al. 2019; Richter 2017, 2019a, b) as they link structural with spectral properties and allow to differentiate between amplifiers and evolutionary graphs in general. Moreover, the findings of this paper underline previous empirical results showing that graphs with different structures can frequently be distinguished by the shape of their spectral density (Gu et al. 2016).

## Conclusions

We have studied the performance and the behavior of an iterative process for identifying transient amplifier for dB updating. Transient amplifiers are networks representing population structures which shift the balance between natural selection and random drift. They are highly relevant for understanding the relationships between spatial structures and evolutionary dynamics. The iterative process implies dynamic graph structures as we consecutively remove edges from regular input graphs. We use the concept of spectral dynamics for analyzing the edge removal process connected with the algorithmic search for transient amplifiers. Our results particularly showed that the spectral dynamics of edge removals finally leading to transient amplifiers are distinct and thus enable differentiation. Thus, we add to answering the question of what structural and spectral characteristics transient amplifier have and how these characteristics can be achieved by edge deletion from a regular graph. Moreover, the results of analyzing the spectral dynamics flow back to the algorithmic process as structural and spectral properties are usable for informing and guiding the process, particularly as the variety of possibilities for deleting an edge from a graph grows massively and therefore needs to be pruned due to computational constraints.

As discussed above the problem of identifying and analyzing transient amplifiers is important for understanding the relationships between spatial structure and evolutionary dynamics. It thus has substantial relevance for real biological processes, as for instance shown for cancer initiation and progression, ageing of tissues, spread of infections and microbial evolution of antibiotic resistance. On the other hand, our topic is also related to a fundamental mathematical question in graph theory which is the relationships between graph spectra and graph structure. Thus, the problems discussed in this paper are also interesting from a graph-theoretical point of view. They contribute to improving our understanding of how edge manipulations are related to spectral properties and reflect upon similarities and differences between the spectra of the normalized and the standard Laplacian.

## Figures and Tables

**Fig. 12:**
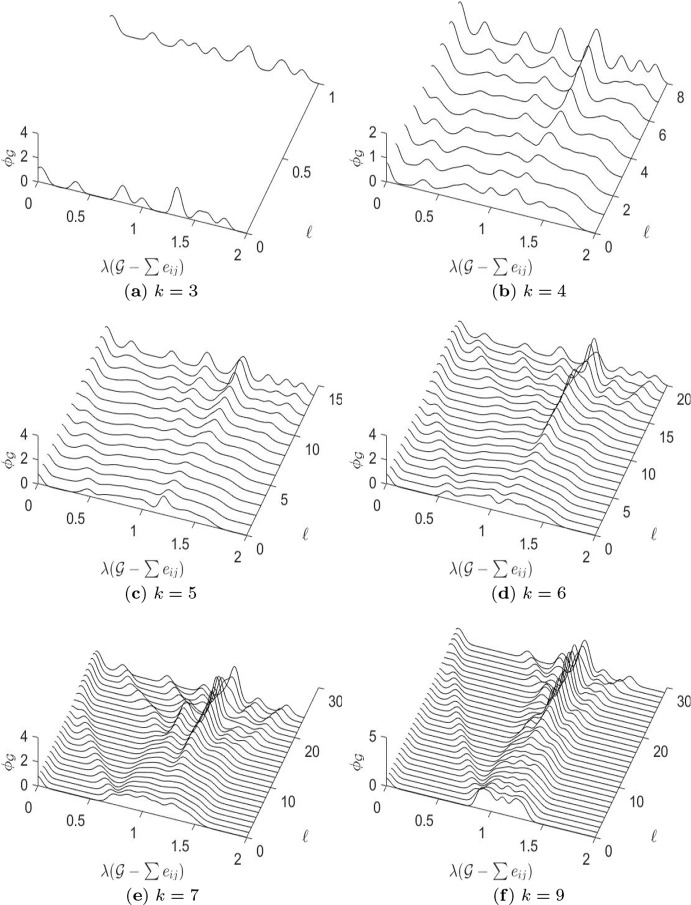
Spectral dynamics expressed by the spectral density $\phi _\mathcal {G}$ describing graph evolutions towards transient amplifiers for order $N=12$ and different *k*. Supplement to Fig. 5

**Fig. 13:**
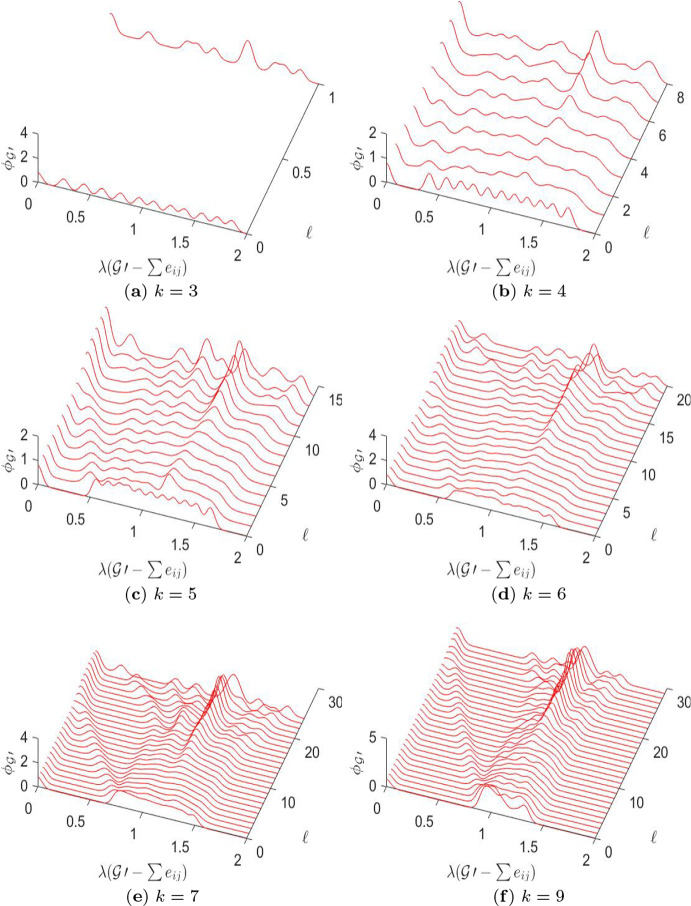
Spectral dynamics expressed by the spectral density $\phi _{\mathcal {G}^\prime }$ describing graph evolutions not towards transient amplifiers for order $N=12$ and different *k*. Supplement to Fig. 5

**Fig. 14:**
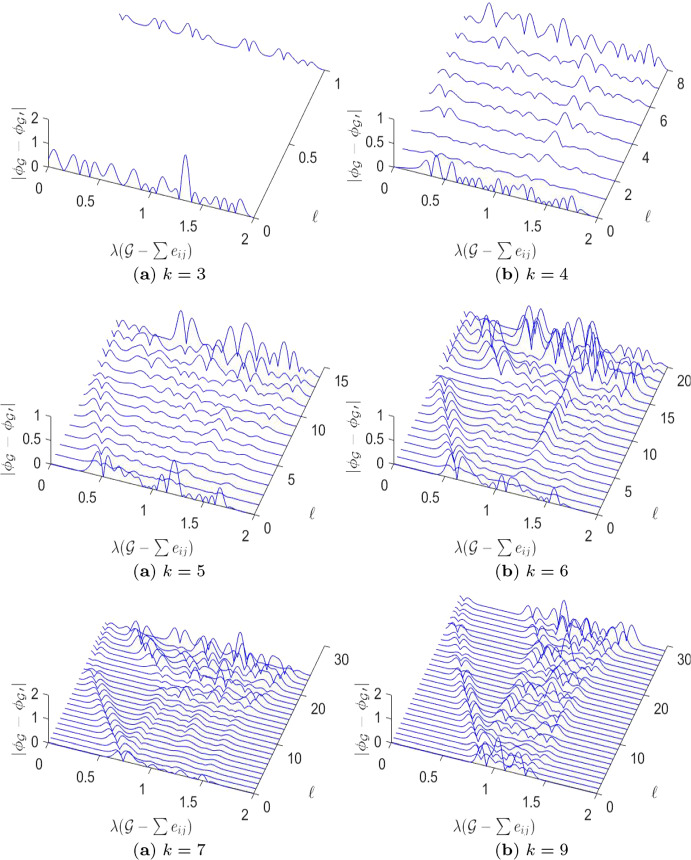
Spectral dynamics expressed by $|\phi _\mathcal {G}-\phi _{\mathcal {G}^\prime }|$ describing the difference between $\phi _\mathcal {G}$ and $\phi _{\mathcal {G}^\prime }$ for order $N=12$ and different *k*. Supplement to Fig. 5

**Fig. 15:**
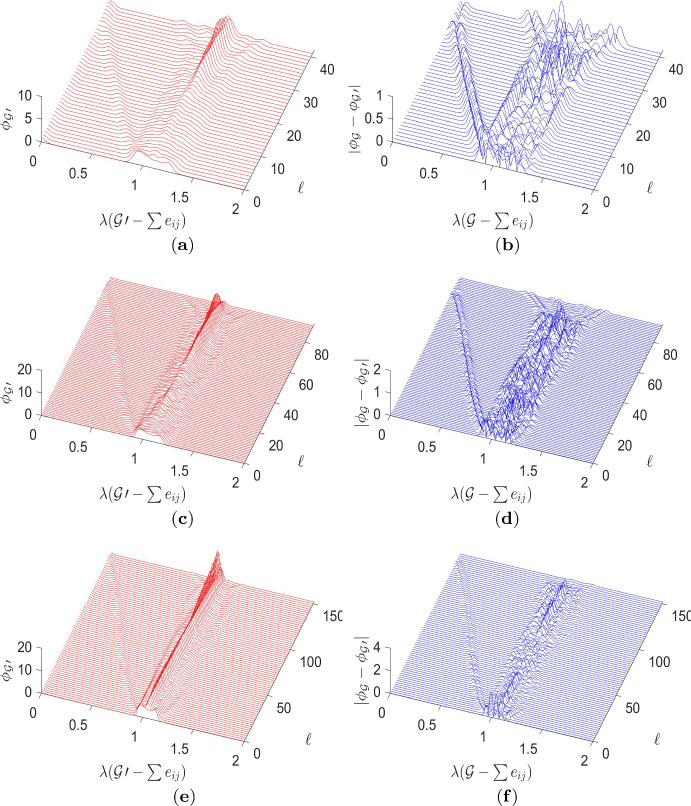
Behaviour of the approximative search of Algorithm 1 for $N=\{14,20,26\}$ and $k=\{11,17,23\}$. The spectral density $\phi _{\mathcal {G}^\prime }$ describing graph evolutions not leading to transient amplifiers and the quantity $|\phi _\mathcal {G}-\phi _{\mathcal {G}^\prime }|$ describing the difference between $\phi _\mathcal {G}$ and $\phi _{\mathcal {G}^\prime }$. Supplement to Fig. 7

## Data Availability

The results of this paper are calculated and visualized with MATLAB. The adjacency matrices of the set of all transient amplifier as well as code to produce the results are available at https://github.com/HendrikRichterLeipzig/IterativeTransientAmplifiers.

## Appendix

### Supplemental figures

See Figs. 12, 13, 14, and 15.

## References

[CR1] Adlam B, Chatterjee K, Nowak MA (2015). Amplifiers of selection. Proc R Soc A.

[CR2] Alcalde Cuesta F, González Sequeiros P, Lozano Rojo Á, Vigara Benito R, Rojas I, Ortuño F (2017). An accurate database of the fixation probabilities for all undirected graphs of order 10 or less. Bioinformatics and biomedical engineering. IWBBIO 2017. LNCS 10209.

[CR3] Allen B, Lippner G, Chen YT, Fotouhi B, Momeni N, Yau ST, Nowak MA (2017). Evolutionary dynamics on any population structure. Nature.

[CR4] Allen B, Lippner G, Nowak MA (2019). Evolutionary games on isothermal graphs. Nat Commun.

[CR5] Allen B, Sample C, Jencks R, Withers J, Steinhagen P, Brizuela L, Kolodny J, Parke D, Lippner G, Dementieva YA (2020). Transient amplifiers of selection and reducers of fixation for death–Birth updating on graphs. PLoS Comput Biol.

[CR6] Allen B, Sample C, Steinhagen P, Shapiro J, King M, Hedspeth T, Goncalves M (2021). Fixation probabilities in graph-structured populations under weak selection. PLoS Comput Biol.

[CR7] Arvind V, Torán J (2005). Isomorphism testing: perspectives and open problems. Bull Eur Assoc Theor Comput Sci.

[CR8] Atay FM, Tuncel H (2014). On the spectrum of the normalized Laplacian for signed graphs: interlacing, contraction, and replication. Linear Algebra Appl.

[CR9] Babai L, Sirakov B, Ney de Souza P, Viana M (2019). Groups, graphs, algorithms: the graph isomorphism problem. Proceedings of International Congress of Mathematicians (ICM 2018).

[CR10] Banerjee A, Jost J (2008). On the spectrum of the normalized graph Laplacian. Linear Algebra Appl.

[CR11] Banerjee A, Jost J (2009). Graph spectra as a systematic tool in computational biology. Discrete Appl Math.

[CR12] Banerjee A (2012). Structural distance and evolutionary relationship of networks. Biosystems.

[CR13] Bondy JA, Murty USR (2008). Graph theory.

[CR14] Butler S, Grout J (2011). A construction of cospectral graphs for the normalized Laplacian. Electron J Combin.

[CR15] Cannataro VL, McKinley SA, St Mary CM (2016). The implications of small stem cell niche sizes and the distribution of fitness effects of new mutations in aging and tumorigenesis. Evol Appl.

[CR16] Cannataro VL, McKinley SA, St Mary CM (2017). The evolutionary trade-off between stem cell niche size, aging, and tumorigenesis. Evol Appl.

[CR17] Chan H, Akoglu L (2006). Optimizing network robustness by edge rewiring: a general framework. Data Min Knowl Disc.

[CR18] Chen H, Zhang F (2017). Spectral dynamics of graph sequences generated by subdivision and triangle extension. Electron J Linear Algebra.

[CR19] Chen G, Davis G, Hall F, Li Z, Patel K, Steward M (2004). An interlacing result on normalized Laplacians. SIAM J Discrete Math.

[CR20] Eldan R, Racz MZ, Schramm T (2017). Braess’s paradox for the spectral gap in random graphs and delocalization of eigenvectors. Random Struct Algorithms.

[CR21] Ghosh A, Boyd S (2006) Growing well-connected graphs. In: Misra P (ed) Proceedings of 45th IEEE conference on decision and control, 2006, pp 6605–6611

[CR22] Ghosh A, Boyd S, Saberi A (2008). Minimizing effective resistance of a graph. SIAM Rev.

[CR23] Gu J, Jost J, Liu S, Stadler PF (2016). Spectral classes of regular, random, and empirical graphs. Linear Algebra Appl.

[CR24] Hindersin L, Traulsen A (2015). Most undirected random graphs are amplifiers of selection for birth–death dynamics, but suppressors of selection for death-birth dynamics. PLoS Comput Biol.

[CR25] Hindersin L, Werner B, Dingli D, Traulsen A (2016). Should tissue structure suppress or amplify selection to minimize cancer risk?. Biol Direct.

[CR26] Hindersin L, Wu B, Traulsen A, Garcia J (2019). Computation and simulation of evolutionary game dynamics in finite populations. Sci Rep.

[CR27] Hoffman C, Kahle M, Paquette E (2019). Spectral gaps of random graphs and applications. Int Math Res Not.

[CR28] Jamieson-Lane A, Hauert C (2015). Fixation probabilities on superstars, revisited and revised. J Theor Biol.

[CR29] Komarova NL, Sengupta A, Nowak MA (2003). Mutation-selection networks of cancer initiation: tumor suppressor genes and chromosomal instability. J Theor Biol.

[CR30] Komarova NL (2006). Spatial stochastic models for cancer initiation and progression. Bull Math Biol.

[CR31] Krieger MS, Denison CE, Anderson TL, Nowak MA, Hill AL (2020). Population structure across scales facilitates coexistence and spatial heterogeneity of antibiotic-resistant infections. PLoS Comput Biol.

[CR32] Li G, Hao ZF, Huang H, Wei H (2018). Maximizing algebraic connectivity via minimum degree and maximum distance. IEEE Access.

[CR33] Lieberman E, Hauert C, Nowak MA (2005). Evolutionary dynamics on graphs. Nature.

[CR34] McAvoy A, Allen B (2021). Fixation probabilities in evolutionary dynamics under weak selection. J Math Biol.

[CR35] Mehatari R, Banerjee A (2015). Effect on normalized graph Laplacian spectrum by motif attachment and duplication. Appl Math Comput.

[CR36] Meringer M (1999). Fast generation of regular graphs and construction of cages. J Graph Theory.

[CR37] Monk T (2018). Martingales and the fixation probability of high-dimensional evolutionary graphs. J Theor Biol.

[CR38] Monk T, Green P, Paulin M (2014). Martingales and fixation probabilities of evolutionary graphs. Proc R Soc A.

[CR39] Möller M, Hindersin L, Traulsen A (2019). Exploring and mapping the universe of evolutionary graphs identifies structural properties affecting fixation probability and time. Commun Biol.

[CR40] Nowak MA, Michor F, Iwasa Y (2003). The linear process of somatic evolution. Proc Natl Acad Sci.

[CR41] Ohtsuki H, Pacheco JM, Nowak MA (2007). Evolutionary graph theory: breaking the symmetry between interaction and replacement. J Theor Biol.

[CR42] Ottino-Löffler B, Scott JG (2017). Evolutionary dynamics of incubation periods. eLife.

[CR43] Ottino-Löffler B, Scott JG, Strogatz SH (2017). Takeover times for a simple model of network infection. Phys Rev E.

[CR44] Pattni K, Broom M, Silvers L, Rychtar J (2015). Evolutionary graph theory revisited: when is an evolutionary process equivalent to the Moran process?. Proc R Soc A.

[CR45] Pavlogiannis A, Tkadlec J, Chatterjee K, Nowak MA (2017). Amplification on undirected population structures: comets beat stars. Sci Rep.

[CR46] Pavlogiannis A, Tkadlec J, Chatterjee K, Nowak MA (2018). Construction of arbitrarily strong amplifiers of natural selection using evolutionary graph theory. Commun Biol.

[CR47] Read RC, Wilson RJ (1998). An atlas of graphs.

[CR48] Richter H, Engelbrecht AP (2014). Recent advances in the theory and application of fitness landscapes.

[CR49] Richter H (2017). Dynamic landscape models of coevolutionary games. Biosystems.

[CR50] Richter H (2019). Properties of network structures, structure coefficients, and benefit-to-cost ratios. Biosystems.

[CR51] Richter H (2019). Fixation properties of multiple cooperator configurations on regular graphs. Theory Biosci.

[CR52] Richter H (2021). Spectral analysis of transient amplifiers for death–birth updating constructed from regular graphs. J Math Biol.

[CR53] Shine A, Kempe D, Liu L, White R (2019). Generative graph models based on Laplacian spectra?. WWW’19: the world wide web conference.

[CR54] Sydney A, Scoglio C, Gruenbacher D (2013). Optimizing algebraic connectivity by edge rewiring. Appl Math Comput.

[CR55] Tkadlec J, Pavlogiannis A, Chatterjee K, Nowak MA (2019). Population structure determines the tradeoff between fixation probability and fixation time. Commun Biol.

[CR56] Tkadlec J, Pavlogiannis A, Chatterjee K, Nowak MA (2020). Limits on amplifiers of natural selection under death–Birth updating. PLoS Comput Biol.

[CR57] Tkadlec J, Pavlogiannis A, Chatterjee K, Nowak MA (2021). Fast and strong amplifiers of natural selection. Nat Commun.

[CR58] van den Heuvel J (1995). Hamilton cycles and eigenvalues of graphs. Linear Algebra Appl.

[CR59] van Nimwegen E, Crutchfield JP (2000). Metastable evolutionary dynamics: crossing fitness barriers or escaping via neutral paths?. Bull Math Biol.

[CR60] Vermeulen L, Morrissey E, van der Heijden M, Nicholson AM, Sottoriva A, Buczacki S, Kemp R, Tavar S, Winton DJ (2013). Defining stem cell dynamics in models of intestinal tumor initiation. Science.

[CR61] Wang J, Huang Q, Belardo F, Marzi EML (2009). A note on the spectral characterization of dumbbell graphs. Linear Algebra Appl.

[CR62] Wills P, Meyer FG (2020). Metrics for graph comparison: a practitioner’s guide. PLoS ONE.

[CR63] WolframMathWorld: regular graphs. https://mathworld.wolfram.com/RegularGraph.html. Accessed 24 Apr 2022

[CR64] Zhang F, Chen YC, Chen Z (2009). Clique-inserted-graphs and spectral dynamics of clique-inserting. J Math Anal Appl.

